# Millennials as a Demographic Bridge to Diversity? Segregation and Diversity of Young Adult Neighborhoods

**DOI:** 10.1007/s11113-025-09954-2

**Published:** 2025-05-12

**Authors:** Noli Brazil, Jennifer Candipan

**Affiliations:** 1 Department of Human Ecology, University of California, Davis, CA USA; 2 Department of Sociology, Brown University, Providence, RI USA

**Keywords:** Segregation, Millennials, Diversity, Young adults, Generation, Neighborhood

## Abstract

As young adults, the Millennial generation emerged as the largest and most racially and ethnically diverse generation in U.S. history. These unique demographic characteristics, along with more progressive self-reported views on racial and ethnic issues, prompted some to label this generation as a demographic bridge to America’s diverse future. This article examines whether these unique characteristics translate into greater neighborhood racial diversity and integration. Specifically, this study sets out to answer whether the neighborhoods where Millennial young adults live are more racially and ethnically diverse and situated in less segregated metropolitan areas than those where young adults from prior generations resided. Using 1990–2020 Census data, we find that young adult Millennials are living in less segregated neighborhoods than their counterparts from previous generations. This pattern holds whether examining the segregation of White young adults from the total population or restricting the analysis to segregation solely among young adults. We further find that a greater presence of White young adult Millennials is positively associated with neighborhood diversity. However, our decomposition analysis, which disaggregates segregation to the agegroup level, suggests that increased uneven sorting among Late Millennial young adults is also driving racial imbalances within neighborhoods among younger and older age groups.

## Introduction

One of the most important demographic trends in recent U.S. history is the emergence of the Millennial generation. Millennials—defined here as those born between 1981 and 2000—are larger in size compared to Generation X and the post-war Baby Boomers, with a population of over 75 million (Frey, 2018a, 2018b; Lee, 2022). Millennials are also more racially and ethnically diverse than prior generations, with nearly 30 percent reporting Hispanic, Asian, and multiracial backgrounds, and more than 40 percent reporting nonwhite overall. In comparison, young adults in 1980 were only 22 percent nonwhite, with Black people representing more than half of this group. Millennials are also more likely to be single, college educated, and living in a city (Frey, 2018a, 2018b; Lee, 2020; Pew Research Center, 2019).

While Millennials are distinct across a variety of demographic traits compared to earlier generations, it is their unique values and preferences that have received considerable attention. Millennials are often portrayed in popular media as progressive and socially liberal (Milkman, 2017; Stoddard, 2010). Survey results show that Millennials exhibit greater broad-mindedness and tolerance, particularly on social issues concerning abortion, same-sex marriage, interracial marriage, immigration, nontraditional family arrangements, and marijuana legalization (Pew Research Center, 2018). Given its combination of nontraditional perspectives and ethnoracial diversity, the Millennial generation has been described as a bridge that could “close the racial and cultural generation gap that, as recent politics have shown, is dividing the nation” (Frey, 2018a, 2018b:4).

An important component to closing this gap is reducing residential racial segregation. The separation of where people live by race and ethnicity is an enduring feature of the American metropolitan landscape. Since peak segregation in the 1960s, Black-White segregation by some measures declined through 2010, albeit with levels remaining stubbornly high, while Hispanic-White and Asian-White segregation levels remained somewhat stable if not slightly increasing during this period (Logan & Stults, 2011). However, at the same time improvements in metropolitan area segregation have stagnated, U.S. neighborhoods are becoming more racially and ethnically diverse (Ellen et al., 2012). These trends suggest that while segregation persists at the aggregate, many individual neighborhoods experience considerable flux in the composition of their populations (Lichter et al., 2017).

Studies of increased diversity, on the one hand, and continued resilience in segregation, on the other, have largely focused on older generations or the total population. However, an empirical analysis of young adult residential settings might reveal diverging patterns from prior generations. A young adult cohort provides a market for radical changes because of its transitory and impressionable nature as its members are transitioning out of their parental homes, experiencing transformative life course events, and being exposed to new ideas while not being old enough to become committed to a career, residence, or family way of life (Rindfuss, 1991). A divergence in the residential patterns of young adults may signal a meaningful transformation, as well as provide insight on future trajectories. The current study contributes to the emerging literature examining the characteristics of the Millennial generation, focusing on the neighborhood racial diversity and segregation patterns among young adults from this generation, while also updating past work with recent data. Specifically, we draw on 1990–2020 decennial census data to address two research objectives: (1) to descriptively examine the relationship between the neighborhood composition of young adult Millennials and neighborhood diversity, and; (2) to descriptively examine young adult Millennial segregation patterns at the metropolitan area level, or put differently, the extent to which young adult Millennials are evenly distributed among neighborhoods within metropolitan areas. We then compare the magnitude of these relationships across young adult cohorts from prior generations. We examine both diversity and segregation because, as others have noted, ethnoracial diversity typically refers to the ethnoracial composition of neighborhoods, whereas segregation typically relates the ethnoracial composition of a larger unit of geography, such as a metropolitan area to its constituent geographies, typically census tracts (Iceland, 2004). Evidence on the link between racial diversity and racial segregation is somewhat mixed, with research finding that community diversification does not always translate to more residential integration (Ellis et al. 2018). Moreover, the presence of diversity in an area can be a potentially fleeting step amid processes of racial turnover, with White households moving out as nonwhite populations progressively increase in representation (Kye & Halpern-Manners, 2023).

In the following section, we summarize the demographic literature on generational change, paying particular attention to the characteristics distinguishing Millennials from previous generations. We then use these differences to help motivate predictions for the segregation and diversity patterns of young Millennials relative to those of prior young adult generations. We do not formally test these mechanisms in the analysis given the aggregated scale of our data, but rather introduce them to help contextualize the differential patterns that emerge from our analysis. The next sections describe the data and methods, followed by a summary of the results. We conclude the paper with a discussion of the implications of the study’s major findings.

## Background

The terms ‘generation’ and ‘birth cohort’ are often used interchangeably in demographic research; however, some argue that a generation implies much more—it refers to groups of people born around the same time period who share a distinctive culture and self-conscious identity by virtue of having experienced the same historical events at roughly the same time in their lives (Ryder, 1985). According to the social identity perspective and generational identity theory, members of a generation have shared value systems that shape boundaries for social and self-categorization, whereby an individual identifies with their generation, potentially leading to the formation of a generational social identity (Mannheim, 1952; Tajfel, 1982; Tajfel & Turner, 2004; Twenge, 2023). The argument here is that even if generations may be difficult to empirically define, they exist as meaningful lived, social identities and are thus worthy of investigation (Milkman, 2017; Twenge, 2023). A key argument justifying the empirical use of generations is that individuals within a generation tend to share similar characteristics, attitudes, and preferences, and these attributes typically differ across generations (Mannheim, 1952). Past studies show a wide range of characteristics differ across generations, including marital status, financial debt, labor force participation, educational attainment, political participation, self-rated health, homeownership and internal migration patterns (Bozick, 2021; Dwyer, 2008; Foster, 2017; Manning, 2020; Milkman, 2017; O’Rand & Hamil-Luker, 2020; Payne, 2022; Tamborini & Iams, 2011; Twenge, 2023).

Generational scholars further argue that, given homogeneity within and heterogeneity across generations, the transition from one generation to another is characterized by major structural societal transformations (Ryder, 1985). A common thread in studies of generations is the importance of significant events that influence the formation of social identities occurring during young adulthood. Significant historical events experienced during young adulthood shape the collective traits and preferences of a generation, and as young adult cohorts enter the job and housing markets and start forming their own households, these traits translate into individual behaviors and lifestyle choices that will collectively shape broader social, economic, and political forces (Manning, 2020; Moos, 2014; Rindfuss, 1991; Ryder, 1985).

### Characteristics of Millennial Young Adults

The largest generation to date is the Millennial generation (Frey, 2018a, 2018b). In addition to its size, scholars have identified several important demographic and socioeconomic characteristics that distinguish Millennials from prior generations. In the most comprehensive demographic overview of Millennials, Frey (2018a, 2018b) found that Millennials, in comparison to prior generations, are more likely to marry later, live in their parental home, and achieve a college degree. Further, Millennials are more likely to be foreign-born, be interracial, marry across race/ethnicity, and speak a language other than English at home (Frey, 2018a, 2018b). Other work also shows that Millennials are less likely to own a car or home during young adulthood, more likely to be in poverty and carry greater debt, and less likely to own a home at age 30 and live in denser housing (Houle & Warner, 2017; Klein & Smart, 2017; Lee, 2022; Pew Research Center, 2019).

One of the most distinguishing features of the Millennial generation is its greater racial and ethnic diversity. Overall, young adult Millennials are 55.8% White (Frey, 2018a, 2018b). In comparison, Generation X and Baby Boomer young adults were 63% and 78% White, respectively (Frey, 2018a, 2018b). Frey concludes that this significant racial and ethnic diversity “will pave the way for the generations behind them as workers, consumers, and leaders in business and government in their acceptance by and participation in tomorrow’s more racially diverse America” (Frey, 2018a, 2018b:4). Although the neighborhood attainment patterns of Millennials were not directly examined, residential racial segregation levels may be lower for the Millennial generation compared to prior generations simply due to its greater racial and ethnic diversity, along with its sheer size (Brazil & Clark, 2017a, 2017b; Sharkey, 2012).

In addition to their demographic characteristics, Millennial attitudes, values, and preferences sharply differ from prior generations (Milkman, 2017). In the most comprehensive documentation of the attitudes of Millennial adults, the Pew Research Center found that Millennials tend to hold more progressive and liberal perspectives relative to prior generations (Pew Research Center, 2018). Most notable, for the purposes of the present study, is the significant gap between Millennials and prior generations in their attitudes towards race and ethnicity. For example, Millennials are more likely than prior generations to report racial discrimination as the main barrier to Blacks’ progress, support racial and ethnic diversity, and advocate for continued efforts towards racial equality. Even among self-reported conservatives, young adult Millennials are more likely than prior generations to agree that Black people are treated less fairly than White people (Pew Research Center, 2018). Some argue that these progressive attitudes suggest optimism for a social agenda focused on inclusion and reducing racial and ethnic inequality (Kaplan, 2020). These attitudes congeal in young adulthood, when a generation mobilizes the wherewithal to translate them into social change (Ryder, 1985).

### Differences in Segregation and Diversity Patterns Across Generations

The unique demographic and attitudinal characteristics of Millennial young adults may help explain differences in diversity and segregation patterns with prior generations. We discuss potential mechanisms underlying differential patterns across generations in this section. Our analysis does not formally test these mechanisms. Instead, we provide a descriptive portrait comparing the residential diversity and segregation patterns of young adults across generations, with the following mechanisms providing context for differences.

If the more progressive attitudes of Millennial young adults influence their residential attainment preferences, then increased racial tolerance is therefore expected to manifest itself in greater receptivity of young people to multiracial communities and neighbors and to occur independently of their socioeconomic and family status. Racial/ethnic differences in residential preferences is an oft-cited factor explaining patterns and drivers of segregation in prior decades (Charles, 2000; Clark, 1992; Krysan et al., 2009). According to this argument, segregation persists largely because members of different racial and ethnic groups simply choose to reside in areas dominated by their own groups. For White households, research consistently shows that they are far less likely than their minority counterparts to express a preference for living in racially mixed or integrated social environments, and that their avoidance of minority neighborhoods stem from negative stereotypes (Bobo & Zubrinsky, 1996; Charles, 2000; Farrell, 2008). This suggests that persistent segregation patterns are largely shaped by their preferences. If more progressive attitudes towards race and greater exposure to nonwhite households in non-residential settings reduce such stereotypes, we may expect White residents to be more open to moving into less White settings (Clark & Brazil, 2019).

Rather than explicit preferences for living in diverse neighborhoods, their greater preference for living in central city neighborhoods may explain increased neighborhood diversity for Millennial young adults. Prior work has shown that compared to prior generations, Millennials are more likely to migrate to cities, and concentrate in centrally located, population dense neighborhoods in the urban core (Lee, 2020, 2022; Lee et al., 2019b, 2024; Myers, 2016). Their higher concentration in cities has been attributed to a combination of preferences for walkable, transit-oriented and amenity-rich neighborhoods, and key demographic and life-cycle differences that would favor more flexible, urban lifestyles, such as lower rates of and later-age entry into marriage and parenthood (Pfeiffer et al., 2019). While a highly educated population is leading the back-to-the-city movement, the average wage increases for this group are outpaced by rising housing costs (O’Regan 2016). Therefore, their choices (or constraints) may lead them to live in lower-resourced urban neighborhoods where housing costs are relatively more affordable. This follows gentrification patterns whereby more socioeconomically advantaged residents, typically White residents, move into spaces where poorer (largely nonwhite) populations had previously occupied (Freeman, 2009). Though not exclusively examining Millennial young adults, Ellen et al. (2012) showed that White in-migration was the primary factor integrating lower-income, primarily renter-occupied, central-city minority neighborhoods, a trend that has rapidly accelerated in recent years. If White residents are leading the Millennial migration back to the city, and their incomes limit their choices to more affordable lower income, minority neighborhoods, this will lead to greater neighborhood diversity levels (Ellen et al., 2013; Owens & Candipan, 2019).

Macro-structural demographic and economic trends may also explain generational differences in segregation and neighborhood diversity levels. In particular, Millennials entered young adulthood during the Great Recession when they were graduating college, entering the labor market, or navigating the early portions of their careers. This critical period of career development was negatively impacted by the poor labor market conditions of the recession and its aftermath, including high unemployment rates, long durations of unemployment, and low wages (Kalleberg & Von Wachter, 2017). These labor market barriers along with decreased housing development also intensified structural housing barriers, thus exacerbating pre-existing housing and rental shortages, and in turn, increasing competition among renters in ways that impacted neighborhood migration patterns (McKernan et al., 2014). As a consequence, Millennials are less well-off economically than members of previous generations in young adulthood, with lower earnings, fewer assets, and less wealth (Kurz et al., 2019). Millennials may then turn to more disadvantaged neighborhoods in urban cores to secure affordable housing options. This migration coincides with concerted efforts by municipal and private actors in many cities to increase population and capital investments in previously disinvested areas (Hall et al., 2015). Urban cores typically house more ethnoracially diverse populations compared to suburban or rural areas. In the case of White young adults, their migration to these neighborhoods would act as drivers of diversity in urban areas, contributing to meaningful reductions in segregation.

A more pessimistic hypothesis is that there would be no (or merely short-lived) changes in segregation and diversity levels. Although Millennials express progressive views when surveyed, their increasingly progressive attitudes towards racial diversity may not necessarily translate into decreased residential segregation if they uphold preferences for same-race neighbors. According to minority group threat theory (Hall and Krysan 2017), White households—at least some subgroups of White households—may be more likely to cluster together in response to the prospect of widespread diversity (South et al., 2011). Although more neighborhoods are experiencing token diversity, and Millennials are expressing a willingness to live in areas with a greater proportion of residents from other race/ethnicities, members of each racial group are still disproportionately concentrated in neighborhoods with same-race neighbors (Howell & Emerson, 2018).

Structural factors may also explain no changes or higher segregation levels. As a consequence of rising housing unaffordability levels, Millennial young adults are more likely than prior generations to move back into their parental homes (VanOrman and Jacobsen, 2020). As such, they will likely live in less diverse, more segregated neighborhoods in comparison to prior generations given the documented patterns of lower diversity and higher segregation for older adults and households with children (Iceland et al., 2010). Furthermore, income and wealth gaps by race and ethnicity are higher for Millennials relative to prior generations, largely attributed to their entrance into the labor market during the Great Recession, suggesting a structural explanation for the persistence of racial inequalities that may map onto spatial patterns of maintained or increased segregation (Frey, 2018a, 2018b; Lee et al., 2024; Milkman, 2017; Myers, 2016). Put differently, one traditional explanation for the persistence of segregation is gaps in economic outcomes between White and nonwhite populations (Charles, 2003). For example, on average, Black households have fewer economic resources to buy into neighborhoods comparable to those occupied by White households (Alba & Logan, 1993); however, even when they do, Black households may not be able to translate their economic capital into lower-poverty White neighborhoods due to racial discrimination in housing, lending, and local zoning laws, as well as Whites’ general resistance to sharing neighborhoods with Black households (Charles, 2003; Logan & Alba, 1993). For White households, unlike for most minoritized households, higher income is arguably associated with residential separation from nonwhite households (Crowder et al., 2012). As a result, we might not observe any differences in segregation and diversity levels across generations, or if differences exist, they might vary across race/ethnicity.

Instead of homogenous patterns across Millennial young adults, key differences between early and late members of the cohort may have led to differential residential attainment patterns. Some argue that the urban revival will subside as the Millennial cohort passes young adulthood and moves out to the suburbs to find employment, form a family, and have children (Myers, 2016; Sharkey, 2012). Distinguishing between Early and Late Millennials, recent studies have found that Early Millennials were moving into suburbs as they entered later adulthood, albeit more slowly than their parents’ generation, but were being replaced by later Millennials (Lee, 2022; Lee et al., 2024). In this case, the more diverse Early Millennial neighborhoods become more segregated over time as they gentrify or experience other neighborhood changes that lower diversity. As Early Millennials transition out of these neighborhoods, Late Millennials fill their vacancies but do so at a time when the neighborhoods are now fully gentrified. This result suggests that Early and Late Millennials are distinct, each facing different sets of constraints and opportunities that, in turn, differently shape their racial and ethnic residential patterns.

## Data and Methods

### Analytic Approach

Our analysis proceeds in multiple steps. We begin with a set of analyses examining generational dynamics of locational attainment at the local scale. Specifically, we descriptively examine neighborhood-level diversity patterns across young adult cohorts. Here, we determine whether Millennial young adults are living in more ethnoracially diverse neighborhoods compared to young adults from prior generations. We address this research objective by performing fixed effects regression models using neighborhood diversity as the outcome and the percentage of residents that are young adults as the main explanatory variable.

We then proceed to investigate generational processes at a higher-level geographic scale, with a focus on racial segregation within metropolitan statistical areas (MSA). We determine whether Millennial young adults are more evenly distributed across neighborhoods (within MSAs) compared to young adults from prior generations. We address this research objective by descriptively analyzing within-MSA neighborhood racial/ethnic segregation patterns by age-group across years. Here, we measure whether segregation levels of young adults differ for Millennials compared to those of prior generations. We then turn our focus to how age-group sorting relates to racial segregation in MSAs. We decompose the total level of segregation in an MSA into its within and between parts to identify how much can be explained by age-group sorting between and within neighborhoods. Here, we examine the segregation levels of young adults relative to the segregation of older age groups and assess their contribution to overall MSA segregation over time. Our results overall will show how the population processes among young adults are associated with neighborhood diversity and segregation, and whether these relationships have changed over time and across generations.

### Defining Generations

Questions about generational differences in locational attainment require that we first define the generational groups, and then identify the focal age cohort within each generation to analyze. Our purpose is not to substantiate empirical definitions of generational periods, but to draw from and utilize commonly understood cohorts to build our argument and analysis. Guided by past work, we categorize generations into Baby Boomers, Gen Xers, and Millennials (Lee et al., 2019b; Lee, 2020; Liu and Reczek, 2021; Pew Research Center, 2019). We define Baby Boomers as those born between 1946 and 1965 while Generation Xers are those born between 1966 and 1980 (Lee, 2022; Lee et al., 2019b; Pew Research Center, 2019). Our primary group of interest is Millennials. Although scholarly research on Millennials is increasing, it is relatively new compared to the literature examining prior generations, and thus the demarcations for group membership are less solidly defined. As such, we follow Lee (2022) and subdivide the Millennial generation into two categories: Early Millennials are those born between 1981 and 1989, while Late Millennials were born between 1990 and 2000.

Our focal age cohort is the 25–29-year-old group in each of our four study years: 1990, 2000, 2010, and 2020. We focused on this period of young adulthood because it comprises a critical life stage that we can compare over time between generational groups. Using this age band further ensures that we do not observe any overlap between generational groups for the 25–29-year-old cohort in any of our study years—e.g., late Gen Xers and Early Millennials do not overlap in 2010; Early and Late Millennials do not overlap in 2020; etc. See Table 1 for a detailed breakdown of these generational groupings, including the birth years associated with each group and years encapsulating young adulthood.

**Table 1:** Overview of generations

Cohort		Birth years	Years ages 25–29	Years ages 35–44	Years ages 45–64	Age in			
						1990	2000	2010	2020
Boomers		*1946–1965*				25–44	35–54	45–64	55–74
	Early boomers	1946–1955	1971–1975 to 1980–1984	1981–1985 to 1990–1994	1991–1995 to 2000–2004	35–44	45–54	55–64	65–74
	Late boomers	1956–1965	1981–1985 to 1990–1994	1991–1995 to 2000–2004	2001–2005 to 2010–2014	*25–34*	35–44	45–54	55–64
Gen X		1966–1980	1991–1995 to 2005–2009	2001–2005 to 2015–2019	2011–2015 to 2025–2029	10–24	*20–34*	30–44	40–54
Millennials		*1981–2000*				1–9	0–19	10–29	20–39
	Early millennials	1981–1989	2006–2010 to 2014–2018	2016–2020 to 2024–2028	2026–2030 to 2034–2038	1–9	11–19	*21–29*	31–39
	Late millennials	1990–2000	2015–2019 to 2025–2029	2025–2029 to 2035–2039	2035–2039 to 2045–2049	––-	0–10	10–20	*20–30*

Shaded cells indicate the study year in which cohorts are young adult age (25–29 years old)

### Neighborhood Diversity

Neighborhood diversity is measured using the entropy index *E*, a common measure of local scale diversity (Wright et al., 2020). We use the census tract as our proxy for neighborhood as it is the geographic unit most used in quantitative research on residential population processes (Iceland & Sharp, 2013). We compute entropy scores for each metropolitan census tract in the U.S. in 1990, 2000, 2010, and 2020.

The population counts used to calculate entropy scores are derived from the 1990, 2000, and 2010 and 2020 decennial census. We combine race and ethnicity data from the census to create the five categories that we use to calculate our entropy score: non-Hispanic White, non-Hispanic Black, non-Hispanic Asian, Hispanic, and other race/ethnicity.

Census tract boundaries change over time. For consistency, we normalize tract boundaries in earlier and later years to 2010 definitions using the Longitudinal Tract Boundary Database (LTDB) (Logan et al., 2014). We further use the 2010 Office of Budget and Management (OMB) definitions for MSAs. Census tract *j* diversity ${E}_{j}$ is calculated as

1 $${E}_{j}= \sum_{r}^{n}{Q}_{r}ln\frac{1}{{Q}_{r}}$$

where ${Q}_{r}$ refers to ethnoracial group *r*’s proportion of a neighborhood population. The formula sets the maximum value of *E* to the natural log of the number of groups *n*. Thus, the maximum entropy in the analysis of five racial groups is ln(5) = 1.61. Such a value would occur only when all groups are of equal size (i.e., each racial group constitutes one-fifth of the population). At the opposite extreme, an *E* value of zero signifies complete homogeneity or no diversity, with all population members in the same group (e.g., an all-White neighborhood). We standardize *E* by dividing it by its five-group maximum and multiplying by 100, thereby setting its range of possible values from zero to 100.

### Metropolitan Area Segregation

Our residential segregation measures further rely on census tract data. We estimate racial/ethnic segregation between neighborhoods within MSAs. We calculate segregation using Theil’s entropy index *H* (Massey & Denton, 1988; Theil, 1972), a structural measure of evenness that can be estimated for dyads or multiple groups, and which compares the level of racial diversity of each tract relative to the racial diversity of the broader MSA. Put another way, *H* captures how (un)evenly racial/ethnic groups are distributed among neighborhoods within MSAs. When scaled from 0 to 100, a value of 0 would indicate no segregation while a value of 100 would describe a metropolitan area without any racial diversity in which each tract is comprised of only one ethnoracial group. A key benefit of using *H*, when estimated for pairwise groups, is its decomposability properties which allow us to identify the within- and between-level components contributing to overall segregation.

Building off the equation used to estimate neighborhood diversity (Eq. 1), *H* can be expressed as the overall difference between tracts’ entropy (${E}_{j}$*)* from the MSA’s entropy ($E$*)*:

2 $$H=\frac{{\sum }_{j=1}^{N}\frac{{t}_{j}}{T}\left(E-{E}_{j}\right)}{E}$$

where *T* represents the total population in the MSA while *t*_*j*_ is the population of tract *j* within that same MSA. We estimate binary *H* between White and minority groups (nonwhite, Hispanic, and when feasible, Black, and Asian), on which we elaborate further below.

While past work often relies on the Dissimilarity Index (*D*) as an easily interpretable segregation measure of evenness, *D* is limited in that it focuses on change occurring above or below racial parity in the overall MSA. On the other hand, *H* estimates these changes along the entire distribution of tracts within an MSA (Fossett, 2017). Moreover, *D* is relatively more sensitive to bias than *H* when estimating segregation with small unit sizes (Fossett, 2017). Unlike other segregation indices (e.g., exposure and isolation indices), *H* is insensitive to changes in the overall racial composition of the MSA, thus facilitating comparisons over time. These stated limitations of *D*, paired alongside the strengths and decomposability properties of Theil’s entropy index, motivated our decision to estimate segregation using *H*.

### Sample Restrictions

We conduct complementary analyses at two geographic scales that first examine population processes underlying neighborhood diversity at the neighborhood level, and then explore residential segregation dynamics at the MSA level. The sample for our neighborhood diversity analysis consists of metropolitan tracts in 1990, 2000, 2010, and 2020. We draw on the same sample for our segregation analysis but must exclude the 1990 study wave because the census does not provide detailed disaggregated age-by-race-by-ethnicity data required to construct our measures. Because small group populations can produce unreliable segregation and diversity measures, we require that census tracts be located within MSAs with at least 1000 White, Black, Hispanic, and Asian residents, following past work (Iceland et al., 2010). We further eliminate zero-population tracts, as well as those with a population of fewer than 500 in any year or with greater than 50 percent of residents living in group quarters (e.g., college dormitories, military barracks). After applying all filters, our final sample includes 57,319 tracts located within 223 MSAs.

### Analysis

We begin the analysis by conducting a detailed examination of the relationship between neighborhood young adult composition and neighborhood diversity within a multivariate context, using fixed-effects regression models. The primary independent variable is the percentage of the tract population that are young adults, which we define as those between the ages of 25 and 29 years old. Note that this measure does not disaggregate by race/ethnicity, but rather encompasses the entire population in that age group. We represent Early and Late Millennial young adults as those between the ages of 25 and 29 years old in 2010 and 2020, respectively. We compare each generational group to those in the same age group in earlier years. By our generational definitions (Table 1), young adult Baby Boomers are represented as 25–29-year-olds in 1990 while young adult Generation Xers are represented as 25–29-year-olds in 2000. The model takes on the following form:

3 $${E}_{tjk}= \mu +year+{\alpha P}_{tjk}+\gamma {P}_{tjk}\times year {+ \beta {X}_{tjk}+{ \rho }_{k}+{\sigma }_{k}t+ \varepsilon }_{tjk}$$

where ${E}_{tjk}$ is diversity of tract *j* in MSA *k* at year *t*, *year* is a set of dummy variables indicating the year (2020 is the reference), ${P}_{tjk}$ is the percentage of residents that are young adults, ${P}_{tjk}\times year$ is the percentage of young adults interacted with year, ${X}_{tjk}$ is a set of tract-level controls, ${\rho }_{k}$ is an MSA fixed effect, ${\sigma }_{k}t$ is a MSA-specific linear trend, and ${\varepsilon }_{tjk}$ is a random error term. The interaction between the year dummy variables and percentage of young adults will test whether the relationship between young adult neighborhood composition and diversity has changed over the last three generations. The tract-level controls include percent nonwhite, log population size, percent poverty, percent of residents using public transportation to commute to work, percent college graduates, percent of employed residents working in professional and related services, percent of occupied units that are renter occupied, percent foreign born, percent of housing units built in the past 10 years, and whether the tract resides in a central city. Robust standard errors are clustered at the MSA level. Tract controls are derived from the long-form decennial summary tape files (for 1990 and 2000) and the 2008–2012 and 2018–2022 American Community Survey estimates (for 2010 and 2020).

These models examine the relationship between diversity and total young adults; however, there may be heterogeneity by race/ethnicity. We next run models that examine the association between neighborhood diversity and the percentage of residents that are White by each age group and year. Here, we determine whether the presence of *White* young adults is associated with changes in residential diversity levels across generations.

Next, we shift our scale to the MSA level for our segregation analysis. Measures of segregation require counts to be disaggregated by race/ethnicity. Segregation operationalized as disparities in residential contact with White households is a convention that dominates the literature, which we also follow for methodological and substantive reasons. We use non-Hispanic White young adults (25–29-year-olds) as our reference group and analyze the extent to which this group is unevenly distributed across neighborhoods relative to 1) the overall population and to 2) other young adults. For our formal analysis, we estimate the racial residential segregation of non-Hispanic White 25–29-year-olds in two ways: 1) in relation to the total population of the comparison group (i.e., Black, Asian, Hispanic, other race/ethnicity); 2) in relation to their age group peers, namely 25–29-year-old nonwhite and 25–29-year-old Hispanic populations. The former determines whether White young adults are segregated from *all* nonwhite residents whereas the latter determines whether White young adults are segregated from *young adult* nonwhite residents. For the latter, we focus on White-nonwhite and White-Hispanic young adult segregation because age-disaggregated counts were included in the initial decennial release and there are sufficient group sizes for each age-band across tracts.^1^

Our residential segregation measures rely on population counts derived from the 2000, 2010 and 2020 decennial census. While the census provides detailed age-disaggregated counts for non-Hispanic White and Hispanic populations, this information is only made available beginning with the 2000 decennial census, thereby limiting our segregation analysis to the study period between 2000 and 2020.^2^ As with the neighborhood entropy scores, our segregation measures are computed using normalized 2010 tract boundaries for each year.

Finally, we turn our focus to how age-group sorting may contribute to racial segregation in MSAs. Are Millennial young adults, for example, sorting differently by race into separate neighborhoods in a way that is different than other generational groups, thereby contributing to overall segregation? We perform a decomposition that examines the extent to which the overall racial segregation within MSAs that occurs via age-sorting could be attributed to sorting between versus within neighborhoods. Here, we can think of the total amount of racial segregation in the MSA as being decomposable into two levels. On one level, total segregation can be explained by different racial/ethnic age groups concentrating into separate tracts. Consider that each tract within an MSA is comprised of several age groups (e.g., Late Millennials, GenXers, etc.). At this first level, we are observing, for example, White-Hispanic segregation between all "tract-age groups" within an MSA. On a second level, the remaining portion of total racial segregation is due to separation, by race, among different age groups within tracts. For example, within a given tract, White Late Millennials may be overrepresented while nonwhite GenXers may also be overrepresented. Both would contribute to racial imbalances due to age-group sorting within the neighborhood. Results from our decomposition analysis indicate whether White and Hispanic young adult Millennials are living in increasingly separate neighborhoods from both each other, as well as whether they are living in increasingly separate neighborhoods from White and Hispanic people from older generations compared to young adults from earlier decades.

To perform our decomposition, we focus on a subset of the population, arraying our data such that each line represents one of five age cohorts (under 18, 25–29, 35–44, 45–64, and 65 and up)^3^ in a given tract within an MSA (i.e., unique at the age-tract-MSA level where age cohorts are nested within tracts which are nested within MSAs). We then aggregate racial/ethnic and overall population counts at each of these nested levels, and for each year separately. Next, we calculate the total levels of White-nonwhite and White-Hispanic segregation (using Theil’s entropy *H*) between age cohorts within MSAs. We then decompose the total MSA segregation into its within and between parts by first calculating racial segregation via age-sorting between neighborhoods, and second, by calculating racial segregation via age-sorting within neighborhoods. These steps allow us to identify how much total segregation can be explained by age-group sorting between and within neighborhoods.

For all analyses, we perform a parallel set of models for two additional age groups in each year—35–44-year-olds and 45–64-year-olds– to examine whether their segregation patterns differ from those of Millennial young adults, as well as young adults from prior generations.^4^ We also make age-group comparisons to explore the degree to which young adult generational trends reflect age-based effects and are distinct from period-based residential patterns affecting *all* age groups. We chose these older age group categories, which contain age ranges that are not equivalent, to minimize overlap between generational cohorts in any year, and to align with prior work that characterizes the 35–44 and 45–64 age groups as mid-life and middle age, respectively (Lee, 2020, 2022). Because these descriptive comparisons are not intended as formal tests of age, period and cohort effects, given the methodological difficulties of conducting such analyses (Yang & Land, 2013), we provide the older-age-group results in the appendix, briefly referring to them in the main text to contextualize the young-adult-age-group trends.

## Results

### Neighborhood Diversity

Our first research objective is to determine whether there are generational differences in the relationship between young adult composition and neighborhood diversity. Specifically, we answer the question: are Millennial young adults living in more diverse neighborhoods than young adults from prior generations? Table 2 shows descriptive statistics for all measures included in our analysis across all study years. Mean neighborhood diversity, as measured by *E*, increases substantially over time, from 30.50 in 1990 to 53.99 in 2020. As well documented, the share of the non-Hispanic White population in our sample declines over time, representing just under 75 percent of the tract population in 1990 but less than 56 percent in 2020. On the other hand, the representation of Black, Asian, and especially Hispanic populations, increases during this same period.

**Table 2:** Descriptive statistics of analysis sample

	1990		2000		2010		2020		Total	
	Mean	SD	Mean	SD	Mean	SD	Mean	SD	Mean	SD
**Neighborhood diversity**										
Multi-group entropy	30.48	20.12	39.72	21.99	45.65	21.54	53.99	20.30	42.46	22.68
*Age composition*										
% 25–29 years old	8.77	3.14	6.99	3.14	6.99	3.31	7.14	3.60	7.47	3.39
% 35–44 years old	15.39	3.18	16.16	2.78	13.21	2.49	12.66	2.41	14.35	3.10
% 45–64 years old	18.76	4.57	22.18	5.03	26.83	5.63	25.77	4.38	23.39	5.86
**Neighborhood characteristics**										
% Non-Hispanic white	74.88	28.52	67.24	30.42	61.32	30.73	55.69	29.29	64.78	30.59
% Hispanic	9.20	16.55	12.62	19.38	16.35	21.30	18.73	21.49	14.22	20.11
% Non-Hispanic black	12.17	22.69	13.58	23.22	14.42	22.95	14.20	21.41	13.59	22.60
% Asian	2.98	6.60	4.00	7.79	5.05	9.09	6.12	10.20	4.54	8.61
% Foreign born	8.60	11.69	11.84	13.95	13.68	14.33	14.37	14.18	12.12	13.76
Population	3520	1556	3957	1542	4286	1945	4601	2494	4091	1965
% Poverty	12.57	11.60	12.51	10.82	15.56	12.39	30.00	17.42	17.66	15.15
% College degree	20.98	15.14	24.80	17.37	28.60	18.91	34.07	20.17	27.11	18.63
% Professional occupation	26.14	12.28	33.14	14.23	35.03	15.39	12.24	6.55	26.64	15.44
% Renter	35.42	22.47	34.51	23.21	36.04	23.25	32.84	21.44	34.70	22.63
% Residents moved in last 10 yrs	64.09	14.75	64.67	13.34	61.23	13.89	59.97	13.62	62.49	14.05
% Housing built < 10 yrs	22.25	20.96	15.99	17.14	11.54	13.40	2.42	4.92	13.05	16.92
% Using public transportation	6.33	12.30	6.23	12.00	6.48	12.66	5.21	10.85	6.06	11.98

Analytic sample consists of 57,319 tracts (2010 boundaries) in 223 metropolitan statistical areas (2010 definitions). Roughly 42 percent of tracts are located in central cities

Younger generations may be more likely to live in racially diverse neighborhoods due to broad demographic shifts driving increases in overall diversity, which create a "floor" of diversity in many areas (structural mobility). On the other hand, increases (or decreases) in neighborhood diversity may be driven by changes in the relative diversity of neighborhoods, independent of these broader population shifts (exchange mobility) (Zhang & Logan, 2016). To set the context for our fixed effects regression models, we first present a descriptive overview of how neighborhood racial diversity among 25–29-year-olds has tracked alongside the overall trend towards greater diversity over time in the U.S.

**Fig. 1:**
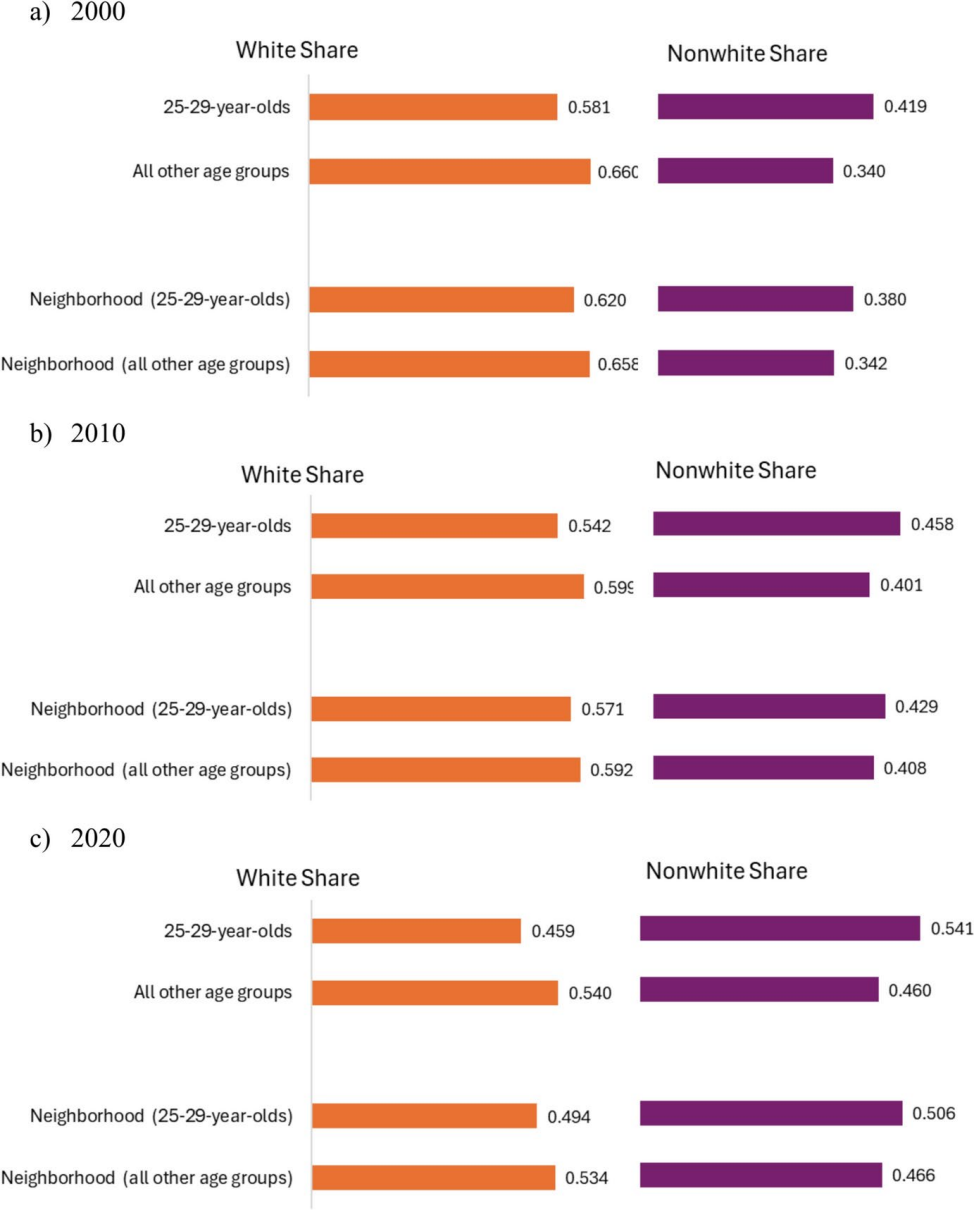
Total population and neighborhood proportion of White and Nonwhite young adults and all other age groups (2000–2020). *Note*: All other age groups refers to all individuals except for 25–29-year-olds. Total neighborhood population refers to the resident population of all age groups except for 25–29-year-olds

Figure 1 shows that the racial composition of 25–29-year-olds in the U.S. population has become increasingly diverse over time, with the proportion of White young adults decreasing from 0.581 in 2000 to 0.459 in 2020, outpacing the decline in the proportion of White individuals in the rest of the population during the same period. This trend among young adults, indicative of broader structural mobility, has naturally led to more racially diverse neighborhoods for this age group. The decline in the average neighborhood proportion of White residents among 25–29-year-olds mostly aligns with population-level trends, reflecting structural mobility where increased neighborhood diversity is a direct consequence of the broader demographic shifts in the young adult population. However, while young adults have experienced an increase in neighborhood diversity over time, they still tend to live in neighborhoods with a slightly higher proportion of White residents compared to their overall population share. Additionally, the proportion of White young adults in neighborhoods declined at a somewhat similar rate to that of the overall young adult population, though slightly faster, and this decline was somewhat larger than the decline observed in the total neighborhood population. While these patterns are also largely consistent with structural mobility, potential elements of exchange mobility may also be contributing to the residential patterns of young adults. We build on these descriptive results next by investigating these patterns in a multivariate framework.

We now turn to our series of fixed-effects regressions that examine the association between neighborhood age-group share and neighborhood diversity over time, net of structural changes in the overall racial composition (accounted for by the model’s MSA-specific linear trend). Table 3 shows the results of models regressing neighborhood diversity on the interaction between year and percentage of total and White residents that are 25–29 years old. We first focus on results for the total age group, which are shown in the first column. The main effects for percent 25–29-year-olds and the yearly indicators support prior evidence of how diversity patterns are associated with age and have changed over time: a greater young adult presence is associated with higher diversity levels, and diversity levels have been increasing since 1990. The marginal effects reveal that across all years a greater young adult presence is associated with greater neighborhood diversity. The interaction results indicate that the association did not change in 2000 and 2010, with coefficients that are not statistically significant at conventional levels, but reduced in 2020, with a statistically significant negative coefficient. In 1990, a one-percentage point increase in 25–29-year-olds is associated with a 1.09 increase in diversity. The associations in 2000 and 2010 are 1.21 and 0.95, respectively, with Chi-square tests indicating that these associations are not statistically significant from one another. In 2020, the association is 0.79, which is statistically different from 0, but statistically smaller compared to prior years based on Chi-square tests. That is, although the greater presence of young adults is associated with higher levels of diversity across all generations, this positive association is lower for the Late Millennial cohort.

**Table 3:** Fixed-effects regression results of neighborhood diversity by total and white young adults and year

Variable	Total		White	
	Coef	*p*	Coef	*p*
% 25–29-year-old	1.09	0.00	1.15	0.00
	(0.10)		(0.09)	
**Year**				
2000	8.43	0.00		
	(0.44)			
2010	16.28	0.00	6.09	0.00
	(0.76)		(0.52)	
2020	29.58	0.00	19.79	0.00
	(1.38)		(1.26)	
**Year x age group**				
2000	0.12	0.10		
	(0.07)			
2010	− 0.15	0.07	0.28	0.00
	(0.08)		(0.07)	
2020	− 0.30	0.00	0.33	0.00
	(0.10)		(0.09)	
% Nonwhite	22.18	0.00	29.37	0.00
	(2.42)		(2.53)	
Log population	1.82	0.00	2.10	0.00
	(0.27)		(0.29)	
% poor	− 16.09	0.00	− 18.94	0.00
	(3.76)		(3.29)	
% using public transportation	− 23.83	0.02	− 27.17	0.01
	(10.37)		(10.41)	
% bachelor’s degree	1.19	0.58	− 0.56	0.79
	(2.14)		(2.14)	
% employed in professional	3.91	0.10	6.56	0.01
	(2.39)		(2.62)	
% renter	− 3.76	0.14	− 2.32	0.34
	(2.52)		(2.40)	
% residents moved in past 10 yrs	24.34	0.03	27.85	0.01
	(11.14)		(10.93)	
% foreign born	44.35	0.00	46.12	0.00
	(5.23)		(5.27)	
% housing built < 10 yrs	− 20.40	0.00	− 19.39	0.00
	(2.65)		(2.69)	
Central city	2.88	0.00	2.94	0.00
	(0.73)		(0.75)	

MSA clustered standard errors in parentheses. Limited to tracts in metropolitan areas with 1000 racial and ethnic group members in all years (n = 223). Model includes MSA fixed effects and an MSA-specific linear trend

Results indicate that the greater presence of young adults is associated with greater neighborhood diversity across all generations, but the relationship is weakest for late Millennial young adults. Our next set of analyses determines whether these differences hold when examining White young adults. That is, we answer the question: are *White* young adults from the Millennial generation living in more diverse neighborhoods than *White* young adults from prior generations? The second column in Table 3 shows results from models regressing diversity on the percent of young adult residents that are White. Similar to results for the total young adult population, we find that a greater White young adult presence is associated with greater neighborhood diversity across all years (marginal effects of 1.15, 1.43 and 1.47 of White percent 25–29-year-olds on diversity in 2000, 2010 and 2020, respectively). However, the positive and statistically significant interaction coefficients for 2010 and 2020 indicate that the association increased for Early and Late Millennials, with the marginal effects suggesting that the increase was similar across both Millennial cohorts. These results suggest that White young adult Millennials appear to be diverging from the downward trend in diversity associated with the total young adult population: while total young adult Millennials exhibit similar or lower positive associations with diversity compared to prior young adult cohorts, White young adult Millennials are associated with higher levels of diversity.

**Fig. 2:**
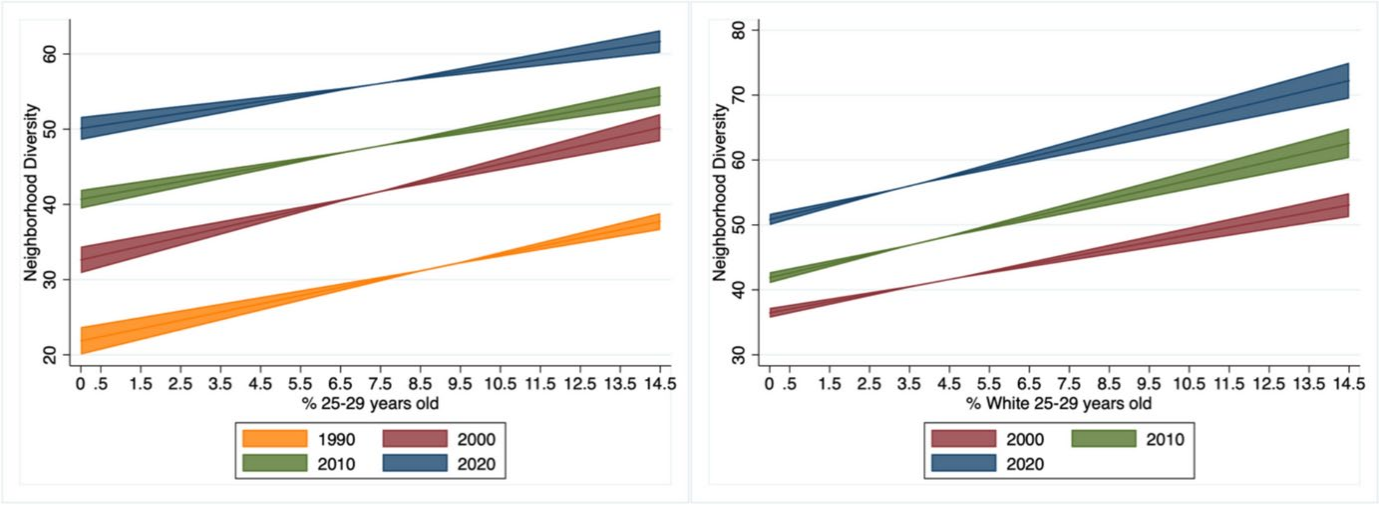
Predicted neighborhood diversity levels and 95% confidence intervals by year for all young adults (left) and White young adults (right). Young adults during the years represent the Baby Boomer (1990), GenX (2000), Early Millennial (2010) and Late Millennial (2020) generations

Marginal effects plots presented in Fig. 2 visually illustrate these patterns. Figure 2 plots the predicted diversity levels and their 95% confidence intervals by year for percent of young adults and percent of young adults that are White. The predicted levels in 2020 are higher in comparison to prior years. However, although positive, the slope is slightly flatter compared to prior years, which aligns with the results presented in Table 3. The proportion of 25–29-year-old residents who are White also support the findings shown in Table 3, with positive slopes in 2010 and 2020 that are steeper compared to the slope in 2000. To account for the possibility that changes in neighborhood diversity are primarily driven by a decline in young adult residents rather than shifts in racial/ethnic composition within young adult neighborhoods, we ran additional models controlling for the proportion of residents aged 35 and 64 years old. We are concerned that neighborhoods with fewer young people are becoming diverse over time, rather than due to neighborhoods with more young people failing to diversify. We found that controlling for older age group composition did not significantly alter the main findings.

**Fig. A1:**
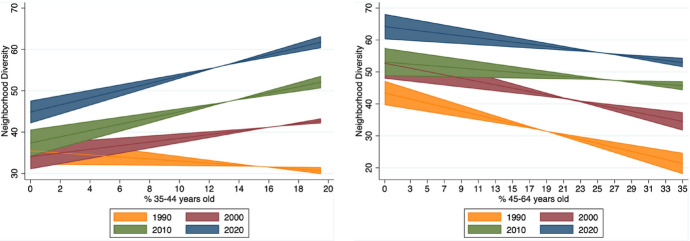
Predicted neighborhood diversity levels and 95% confidence intervals by percent 35–44 years old and 45–64 years old and year

**Fig. A2:**
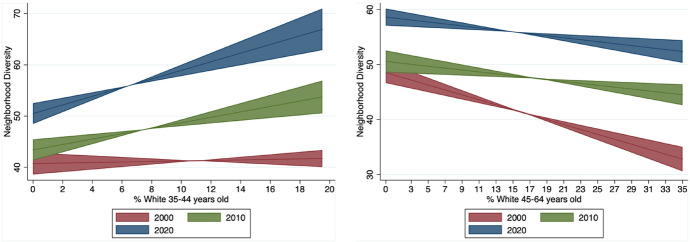
Predicted neighborhood diversity levels and 95% confidence intervals by percent White 35–44 years old and 45–64 years old and year

The differences between the young adult age groups likely reflect generational differences in residential patterns rather than just period-based residential patterns affecting *all* age groups. To further explore this possibility, we perform a parallel set of models that analyze the association between adult composition and neighborhood composition with two other adult age cohorts (35–44-year-olds and 45–64-year-olds), separately and in each year. These results are provided in Appendix Tables A1–A2 and Appendix Figures A1–A2. Both total and White older age groups are associated with lower diversity levels in comparison to young adults across all years. In the case of the 45–64-year-old population, the association is negative or zero in all years. These findings corroborate prior work showing decreasing neighborhood diversity levels for Baby Boomer and Gen X young adults as they age into older adulthood (Sharkey, 2012). This is also the case for Early Millennials, as their greater presence as 35–44 year olds in 2020 is associated with lower diversity levels compared to when they were young adults in 2010. In terms of changes over time, the association between percent 35–44-year-old and diversity increases from 1990 to 2010, and then slightly increases in 2020. For percent 45–64-year-old, the magnitude of the negative association does not change between 1990 and 2000 but reduces in 2010 and then again in 2020. Collectively, our results indicate diversity trends that reflect some age and period patterning, but also unique age-generational and ethnoracial differences. Specifically, Early Millennial young adults experienced greater neighborhood diversity than previous generations, while Late Millennials saw equal or lower diversity levels. However, among White young adults, both Early and Late Millennials experienced increasing neighborhood diversity over time. A comparison of these trends to older age groups reveals that the differences across young adult generations are not merely a by-product of period differences impacting all age groups.

### Metropolitan Area Segregation

**Fig. 3:**
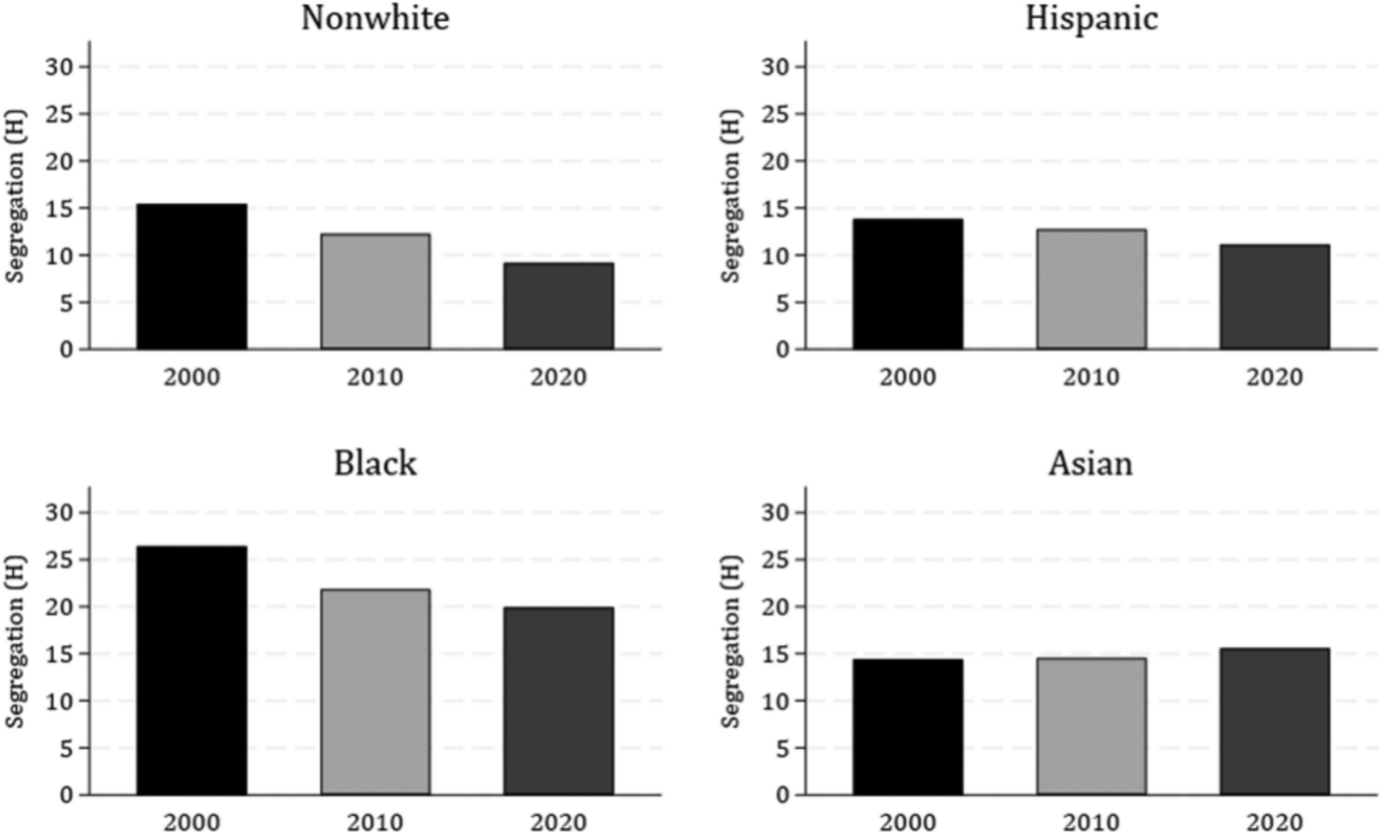
Segregation (H) between 25–29-year-old Whites and Total Population (by Year). *Note*: Segregation is measured using the Theil’s Entropy (H) (scaled 0–100). White, Black, and Asian are all non-Hispanic. n = 223 MSAs. Young adults during the years represent the GenX (2000), Early Millennial (2010) and Late Millennial (2020) generations

Our second research objective compares the segregation patterns of young adults across generations. We first examine White young adult segregation from the total nonwhite population. That is, we answer the question: How evenly distributed are Millennial White young adults and total nonwhite groups in comparison to prior generations? We estimate racial segregation in terms of *H* within MSAs (and between neighborhoods), by year, between all White young adult residents and: 1) all nonwhite; 2) all Hispanic; 3) all Black; and 4) all Asian residents (Table 4). Our tables are accompanied by figures which display the average racial segregation of White 25–29-year-olds (our focal age group) from the overall resident population of each ethnoracial minority group (Fig. 3). (See appendix table A3 for information on trends in total segregation—i.e., pooled for all age groups—over time.)

**Table 4:** Neighborhood racial segregation between white young adults and total population and between young adult peers, 2000–2020

Measure	Year						Δ 2000–2020
	2000		2010		2020		
	Mean	SD	Mean	SD	Mean	SD	
**White 25–29 vs. total population**							
Total Nonwhite	15.46	*8.65*	12.26	*6.66*	9.17	*5.07*	− 6.29
Total Hispanic	13.82	*8.67*	12.76	*7.37*	11.14	*6.49*	− 2.68
Total black	26.42	*13.91*	21.86	*11.91*	19.92	*10.55*	− 6.50
Total Asian	14.41	*6.41*	14.55	*6.67*	15.57	*7.29*	1.16
**Age-specific segregation (25–29 vs. 25–29)**							
White-Nonwhite	18.62	*9.65*	16.05	*8.21*	11.43	*6.25*	− 7.18
White-Hispanic	16.20	*9.38*	15.61	*8.84*	12.83	*7.83*	− 3.37

n = 223 MSAs; Segregation is measured by Theil’s Entropy (*H*) (scaled from 0–100); White, Black, and Asian are all non-Hispanic. Young adults during the years represent the Baby Boomer (1990), GenX (2000), Early Millennial (2010) and Late Millennial (2020) generations. See appendix table A3 for information on overall (pooled for all age groups) segregation trends over time

Segregation estimates of the White young adult population (White 25–29-year-olds) from the total population of each ethnoracial group reveal a consistent pattern: the segregation between White young adults (White 25–29-year-olds) from the total neighborhood population of Hispanic and, especially, Black residents declines over time from 2000 (Gen X) to 2010 (Early Millennial), and again from 2010 to 2020 (Late Millennial) (Fig. 3). Segregation between White young adults and the overall Asian resident population, however, reveals a different pattern. While segregation between White young adults and all Asian residents remained relatively stable between Gen X (in 2000) and Early Millennials (in 2010), it increases slightly for Late Millennials in 2020 (from 14.41 in 2000 to 15.57 in 2020).

We observe the highest segregation levels between millennial White young adults and all Black residents, but also the sharpest declines over time (from 26.42 to 19.92, reflecting roughly half a SD decrease). The decline in segregation between White young adults and Hispanic residents was more modest—from 13.82 in 2000 (GenXers) to 11.14 in 2020 (Late Millennials), reflecting roughly a quarter SD decrease. While somewhat modest in absolute terms, we argue that represents a nontrivial decrease given the durability of segregation in the U.S.

**Fig. A3:**
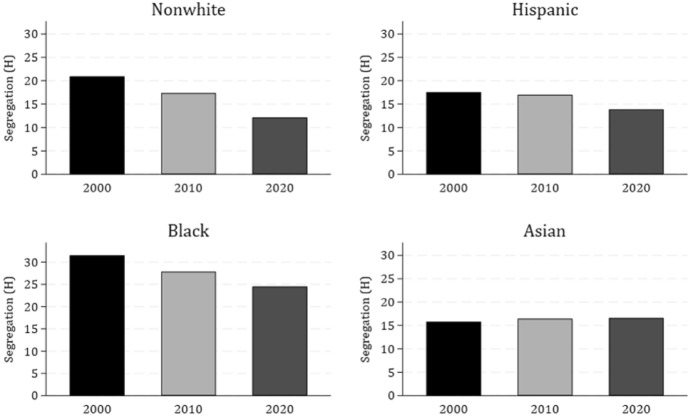
Segregation (H) between 35–44-year-old whites and total population (by year). *Note*: Segregation is measured using the Theil’s Entropy (H) (scaled 0–100). White, Black, and Asian are all non-Hispanic. n = 223 MSAs

**Fig. A4:**
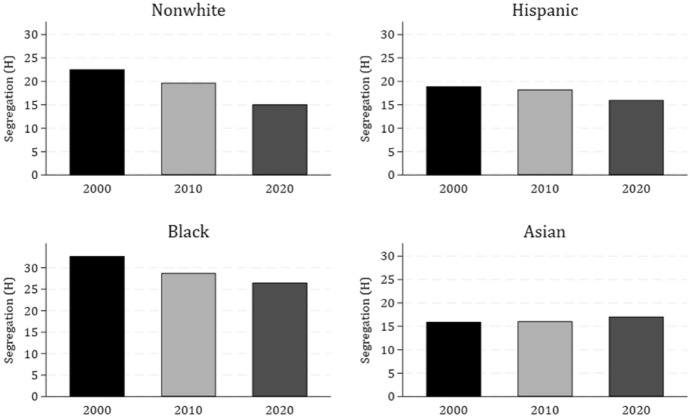
Segregation (H) between 45–64-year-old whites and total population (by year). *Note*: Segregation is measured using the Theil’s Entropy (H) (scaled 0–100). White, Black, and Asian are all non-Hispanic. n = 223 MSAs

Examining segregation levels for older age groups yields similar patterns as those for neighborhood diversity in the prior section. Similar to patterns for young adults, segregation levels for White 35–44-year-olds and 45–64-year-olds also decrease from 2000 to 2020, although the decrease is somewhat larger in absolute terms than the decrease for White 25–29-year-olds (see Appendix Table A3 and Figures A3–A4).^5^ Relative to White young adults, White 35–44-year-olds and 45–64-year-olds tend to be more segregated from all groups (Black, Hispanic, and Asian residents) across all years. This indicates that White Late Boomers, Gen Xers, and Early Millennials tend to reside in neighborhoods that are relatively more separated from Black, Hispanic, and Asian people than White Late Millennials in 2020.^6^

**Fig. 4:**
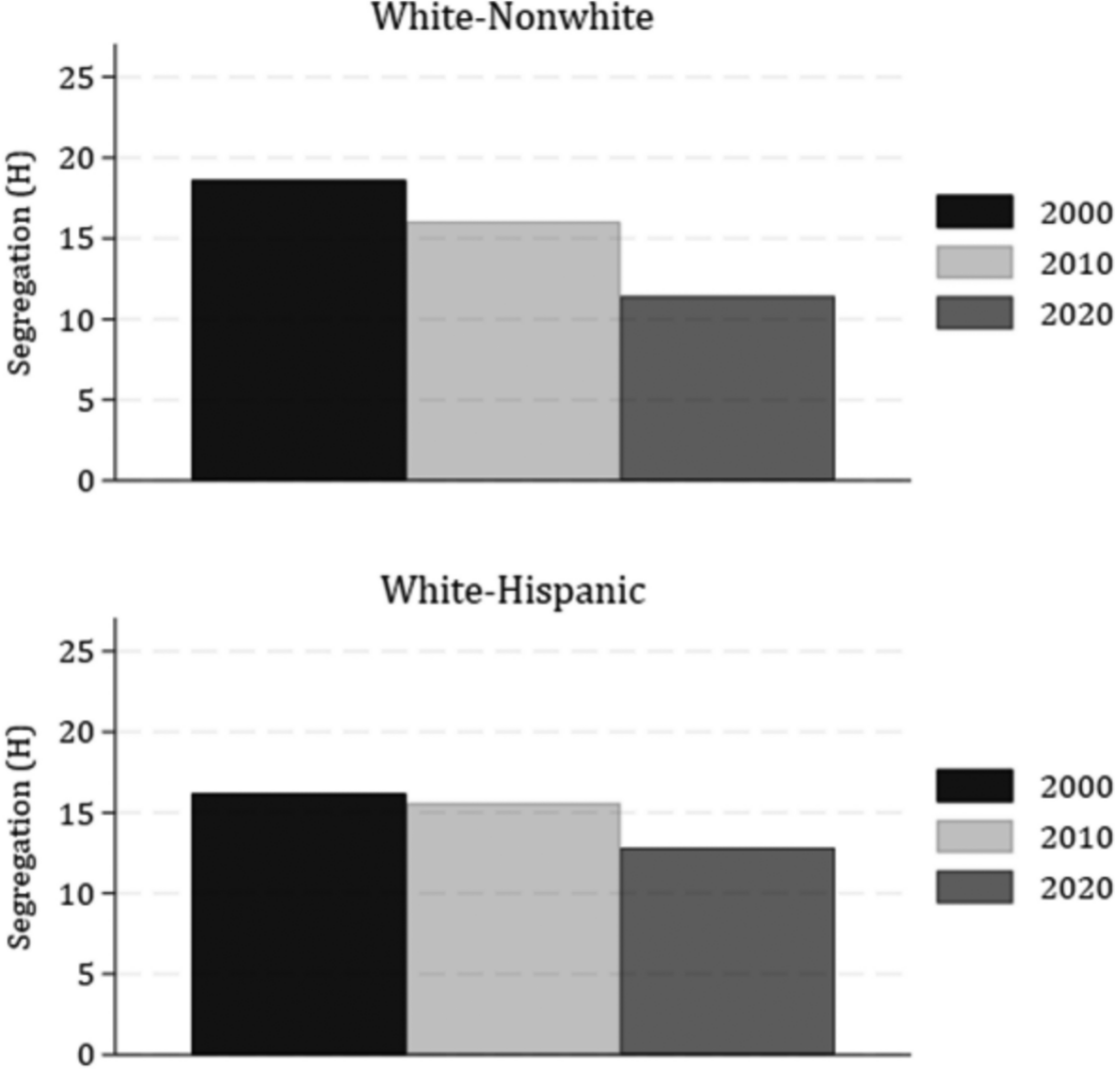
Age-specific (25–29-year-old) White-Nonwhite and White-Hispanic Segregation (H), 2000–2020. *Note*: Segregation is measured using the Theil’s Entropy (H) (scaled 0–100). White, Black, and Asian are all non-Hispanic. n = 223 MSAs. Young adults during the years represent the GenX (2000), Early Millennial (2010) and Late Millennial (2020) generations

Results thus far indicate that segregation of White young adults from all Black and Hispanic residents trends downward for Early Millennials, and especially for Late Millennials, but that Late Millennial White young adults are slightly more separated from Asian residents in 2020 compared to White young adults from previous generations. Our next set of analyses determines whether these trends hold when examining the segregation of White young adults with their same-age peers. That is, we answer the question: How evenly distributed are Millennial White and nonwhite young adults compared to their distribution in prior generations? We calculate age-specific segregation of White young adults from 1) nonwhite and 2) Hispanic young adults in each year (bottom panel of Table 4). We illustrate these results in figures that show the mean neighborhood racial segregation (within MSA) over time (2000 to 2020) that occurs between 1) White and nonwhite and 2) White and Hispanic young adults (Fig. 4).

**Fig. A5:**
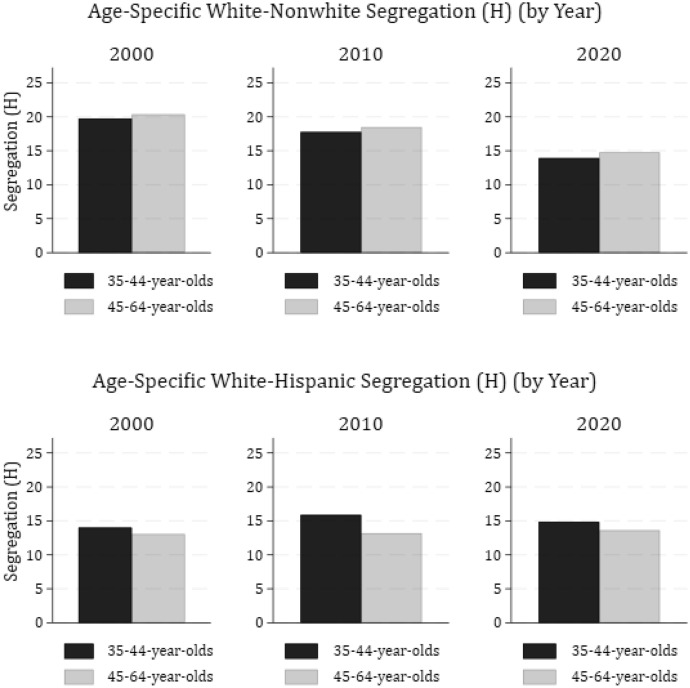
Age-specific white-nonwhite and white-hispanic segregation, 2000–2020. *Note*: Segregation is measured using the Theil’s Entropy (H) (scaled 0–100). White, Black, and Asian are all non-Hispanic. n = 223 MSAs

White-nonwhite segregation among young adults decreases somewhat from 2000 (Gen X) to 2010 (Early Millennial), then decreases much more by 2020 (Late Millennial). Compared to older age groups, White-nonwhite segregation among young adults is relatively lower than segregation among 35–44-year-olds and 45–64-year-olds across all years (see Appendix Table A4 and Appendix Figure A5), indicating that young adults were more evenly distributed by race across neighborhoods with their same-age peers compared to older age groups. This is especially the case for Millennial young adults in 2020, suggesting that segregation dynamics have changed somewhat over the past two decades.

Similar to White-nonwhite young adult patterns, the segregation between White and Hispanic young adults decreases over time, though it declines more steadily between decades (from 16.20 in 2000 to 12.83 in 2020, representing roughly a third SD decrease). This decrease is somewhat larger than the decrease in segregation between White young adults and the overall Hispanic population (compare to Fig. 3). Patterns for young adults differ somewhat compared to patterns observed for older groups (35–44-year-olds and 45–64-year-olds), which revealed a good deal of variation in same-age White-Hispanic segregation levels across all years (compare to Appendix Table A4). Segregation remained rather steady or slightly increased over time among all older age groups. 25–29-year-olds in 2000 (Gen X) and 2010 (Early Millennials) are more segregated from Hispanic young adults than White and Hispanic older adults in these years (compare to Appendix Table A4), indicating that White and Hispanic young adults consistently sorted into separate neighborhoods. However, by 2020, White and Hispanic Late Millennial young adults were slightly less segregated from one another compared to White and Hispanic older adults.

### Segregation Decomposition Analyzing Age-Group Sorting Within MSAs

Finally, we further examine the extent to which racial segregation in metropolitan areas occurs by age group. Table 5 presents results from a decomposition analysis that identifies the relative contributions to total MSA racial segregation that occurs due to differential residential sorting by age groups within and between neighborhoods in 2000, 2010, and 2020.

**Table 5:** Decomposition of MSA segregation (*H*) within and between neighborhoods by age cohort

		2000		2010		2020	
		White-Nonwhite	White-Hispanic	White-Nonwhite	White-Hispanic	White-Nonwhite	White-Hispanic
Total segregation		24.50	19.62	22.75	21.35	18.72	19.90
	Between neighborhoods	20.76	14.54	18.47	15.36	14.64	14.21
	Within neighborhoods	3.74	5.07	4.28	5.99	4.08	5.69
	Proportion explained between neighborhoods	84.74	74.14	81.19	71.95	78.19	71.40

The decomposition analysis was performed on a subset of the sample consisting of five age cohorts within all tracts: the population under 18, 25–29 years, 35–44 years, 45–64 years, and 65 and up. See Table 1 for an overview of how each age cohort maps onto the generations in each year. Mean total segregation refers to residential sorting that occurs between all age groupings within all neighborhoods in the MSA in each year. Segregation between neighborhoods refers to sorting among age groups between tracts. Segregation within neighborhoods refers to sorting that occurs between age groups within tracts. White refers to non-Hispanic White. n = 223 MSAs

Recall that Theil’s *H* index is perfectly additive which allows us to decompose total segregation into its between and within components. In this case, we treat age groups as administrative units that nest within tracts, which further nest within MSAs. Although related to our segregation analyses presented above, our focus here on age-group sorting within and between neighborhoods (within MSAs) is conceptually and empirically distinct in that we analyze the extent to which differential race-based sorting between age groups within and between tracts contributes to total racial segregation in the MSA. Our decomposition shows that overall MSA segregation is explained by uneven sorting among age groups between neighborhoods, rather than differential sorting within neighborhoods, particularly for White-nonwhite segregation, although this relationship has attenuated somewhat over time.

The first row of Table 5 displays the total level of racial segregation that occurs between age groups within MSAs over time. Focusing on White-nonwhite segregation (left column in each year), we see that total White-nonwhite segregation among age groups steadily declines from 2000 to 2020 (from 24.50 to 18.72). The contributions to total MSA White-nonwhite segregation due to age-group sorting also changed slightly over the years. We observe these changes in the second row, which shows how much of the overall racial segregation of age groups within MSAs occurs between tracts. The vast majority of total White-nonwhite segregation by age groups occurs between neighborhoods. However, the contribution to total MSA racial segregation due to age sorting between neighborhoods declines from 2000 and 2020. Nearly 85 percent of total segregation can be explained by age-group sorting between neighborhoods in 2000, but this number decreases to roughly 78 percent in 2020. On the other hand, a relatively smaller amount of total MSA racial segregation among age groups is driven by sorting within neighborhoods (third row), but these contributions have increased somewhat over time (from 15 percent in 2000 to 22 percent in 2020).

Unlike White-Nonwhite residential segregation, overall White-Hispanic segregation among age groups remains stable over time, and actually increases slightly from 2000 to 2020 (from 19.62 in 2000 to 19.90 in 2020). Consistent with White-nonwhite segregation, most of overall White-Hispanic segregation occurs via the differential distribution of age cohorts between neighborhoods rather than within neighborhoods in MSAs, although these relative contributions have declined over time. About 74 percent of total segregation in 2000 can be explained by uneven age-group sorting between neighborhoods, but this number decreases slightly to roughly 71 percent in 2020. Nearly 30 percent of White-Hispanic segregation in 2020 is driven by sorting among age groups within neighborhoods (up from 25.9 percent in 2000 to 29.6 percent in 2020). This result indicates that while White and Hispanic Late Millennial young adults may not be sorting into different neighborhoods from each other to the degree as young adults from prior generations, they are living in more racially imbalanced neighborhoods by age group than earlier years. Combining these findings with our earlier analysis—specifically, that age-specific White-Hispanic segregation among 25–29-year-olds in 2020 (see Table 4 and Appendix Table A4)—suggests that young adult Gen Xers and Early Millennials contributed to rising White-Hispanic overall segregation in earlier decades, as well as growing racial imbalances between age groups within their neighborhoods. Conversely, Late Millennial young adults are potentially contributing to the decrease in the overall segregation between White and Hispanic people.

## Discussion

Growing up as the most racially and ethnically diverse generation in U.S. history, with more progressive views on social issues than prior cohorts, the Millennial generation has been described as a social, economic, and political bridge to America’s diverse future (Frey, 2018a, 2018b). In this study, we set out to examine this claim through the lens of where Millennial young adults live. Specifically, the goal of this study was to investigate whether Millennial young adult neighborhoods are more racially diverse and less segregated compared to the young adult neighborhoods of prior generations.

We found that White young adult Millennials are living in less segregated neighborhoods than White young adults from previous generations. In contrast, we find either smaller or no reductions in segregation for older age groups. These patterns hold whether examining the segregation of White young adults from the total population or restricting the analysis to segregation solely among young adults. We further found the greater presence of White young adult Millennials is positively associated with neighborhood diversity. Collectively, these results provide support for the characterization of Millennials as a bridge to diversity. However, our decomposition analysis disaggregating segregation to the age-group level suggests that the greater uneven sorting among Late Millennial young adults is also driving racial imbalances within neighborhoods among younger and older age groups.

One factor potentially explaining the contrasting patterns between White young adults from different generations is that the very neighborhoods into which both Millennial cohorts are moving are themselves experiencing trajectories of ethnoracial compositional change. Prior work has found that Millennials are more likely to live in urban neighborhoods compared to prior generations. This preference has been attributed to higher levels of educational attainment, greater tendencies to live alone, and their choice to delay traditional life milestones, such as parenthood, in favor of more flexible lifestyles close to amenities (Ehlenz et al., 2020). Young adult Millennials were at the vanguard of the back-to-city movement, and the neighborhoods they were occupying were often in the early stages of gentrification (Ehlenz et al., 2020; Moos, 2016). Neighborhoods undergoing earlier stages of gentrification typically have non-trivial proportions of nonwhite residents before experiencing some level of racial transition as White newcomers move into the community (Owens & Candipan, 2019; Rucks-Ahidiana, 2021). As such, the migration of White young adults into these neighborhoods would act as drivers of diversity in urban areas, contributing to modest but meaningful reductions in segregation. Late Millennials are even more likely to settle in urban neighborhoods than their Early Millennial counterparts (Lee, 2022; Myers, 2016). Our decomposition analysis, which showed that segregation was increasingly explained by age-sorting by White households within neighborhoods over time, corresponds with the view of neighborhood racial transition occurring via the influx of young adult White residents into nonwhite communities.

Another potential explanatory factor relates to the timing of broader macro-structural influences and whether they occurred during critical life stages for members of each generation. For example, we are likely observing cohort differences in the effects of the Great Recession based on when it occurred during each cohort’s life course (Lee et al., 2024). Millennials were graduating from college and entering the workforce during the recession, and thus lacked the job and housing opportunities to advance to the next stages of their employment and housing careers, forcing many to delay marriage and childbearing, with some unable to move to better housing or forced to move back long term into their parental homes (Mawhorter, 2017). The housing crash and subsequent decline in housing development exacerbated pre-existing housing shortages and increased competition among renters (Lens, 2018). Millennials seeking housing in the face of rising housing costs potentially turned to more affordable areas in central city neighborhoods, many of which were predominantly lower income and nonwhite (Lee, 2020; Myers, 2016). City officials welcomed these young White adult movers as they sought to revitalize the urban core, which typically house more ethnoracially diverse populations compared to suburban or rural areas (Pfeiffer et al., 2019). Although the impact of the recession on income and credit gradually lessened as late Millennials entered young adulthood, housing affordability continued to decline in metropolitan areas. As a result, Late Millennials faced similar housing constraints as their predecessors and maintained strong preferences for urban living as they entered young adulthood (Lee et al., 2019a). As a generational cohort, Millennials' preferences for urban living, combined with the economic constraints they faced, may have fostered more integrated neighborhoods in metropolitan cores. These explanations are speculative. Our objective was to provide a descriptive portrait of Millennial segregation and diversity trends as a baseline understanding of these patterns have not been established in prior work. Findings from our descriptive study provide fruitful grounds for future scholarship that can disentangle the mechanisms driving these trends.

Future research should also investigate Millennials’ neighborhood diversity and segregation patterns as they age into older adulthood. The promising trends highlighted in this study may be temporary in that Millennial segregation and diversity patterns may start to mimic prior generations as Millennials start forming families, buying homes, and establishing careers. Prior work has shown that Millennials are following previous generations who were relatively more likely to live in urban neighborhoods at younger ages and then shifted to more suburban living as they got older (Lee et al., 2024). In particular, older Millennials appear to be migrating out of central urban neighborhoods to peripheral suburban areas, seeking affordable or family-sized housing (Lee et al., 2024). Although inner-ring suburbs have experienced rising diversity, outlying suburban neighborhoods have experienced increasing segregation, suggesting that the positive trend in segregation and diversity for Millennials may be muted or reversed as they enter fringe suburban neighborhoods in older adulthood (Lichter et al., 2023).

While the decrease in Millennial White young adult segregation is true in relation to Black and Hispanic total and young adult populations groups, segregation in relation to Asian residents has increased. Specifically, White and Asian young adults have become increasingly segregated over time and across generations. One potential factor explaining increasing young adult White-Asian segregation is rising income disparities between these groups. Some of this was driven by the foreclosure crisis during the Great Recession, translating into increasing segregation when stratified by both race and income (Crowell & Fossett, 2022; Elbers, 2021; Hall et al., 2015; Intrator et al., 2016). Another potential factor is the emerging presence of new Asian destinations in metropolitan areas, which have seen increasing segregation levels during the study’s observation period (Lichter et al., 2010; Park & Iceland, 2011). Kye (2023) documents increasing White flight from Asian ethnoburbs that started out as majority White communities, particularly in higher-SES neighborhoods. The study uses an ethnic stratification perspective to explain this result, which asserts racialization as a mechanism that may lead to White flight and segregation, even in middle-class Asian communities (Kye, 2023). We leave the investigation of the potential factors explaining this rising segregation to future research as it is beyond the scope of this descriptive study. Such research is needed as the Asian population is expected to continue growing through immigration and fertility, and by moving into new destinations across the country (Brazil, 2019; Frey, 2011, 2018b). The increase in segregation between White and Asian populations, along with the persistently high segregation between White and nonwhite, Black and Hispanic groups and the migration of Millennials out of urban areas into potentially more segregated peripheral suburban neighborhoods, highlight the enduring persistence of racial segregation. Results from our decomposition analysis further suggest that young adults continue to experience substantial (and perhaps increasing) racial imbalances in more localized residential contexts.

Our results further raise additional avenues for future research. Our findings are specific to the neighborhood level, and thus cannot be generalized to individual residential decisions. Future work employing micro-level longitudinal data could potentially tease apart the individual-level factors, such as socioeconomic standing and family status, from the structural factors that may be driving segregated young adult residential patterns. Individual-level analyses can further investigate residential mobility patterns influencing changes in neighborhood diversity and segregation, such as distinguishing between young adults moving to more/less diverse neighborhoods versus remaining in neighborhoods that are changing around them. Sufficient individual-level data may also allow for an analysis of young adults that spans a wider age band, as well as an examination of segregation across a longer time period than those represented in this study. Although adopted as a unit of analysis in many social science research applications, the concept of a generation has been criticized for its potentially arbitrary birth-year cut-offs and substantial within variation between members. The use of individual-level data mitigates these critiques.

Our findings center on the patterns of White young adults across generations. Questions remain, however, about whether young adults from other ethnoracial groups follow similar segregation and diversity patterns. Differences are likely present given prior work demonstrating that segregation is primarily driven by White neighborhood attainment patterns (Quillian 2002). Questions also remain about the extent to which the COVID-19 pandemic played a role in Late Millennial young adult patterns. The 2020 decennial census was collected during the height of pandemic, potentially introducing enumeration issues including undercounts of certain groups and increased nonresponse rates.

In addition to future work exploring the potential mechanisms driving increasing segregation among young adults, subsequent research should further investigate its social, political and economic implications. This is important as Millennials age into later adulthood when residential mobility may likely fortify segregation patterns as they start forming families, buying homes, and establishing careers (Brazil & Clark, 2017a, 2017b; Sharkey, 2012). Finally, future work should also begin examining the Gen Z generation as its members start to enter young adulthood. As an even more racially and ethnically diverse generation growing up during a period of significant political division and social change, it is unclear whether the young adult Gen Z cohort may act as a vanguard for greater residential diversity or continue to reinforce the segregation patterns established by its predecessors.

Despite some limitations, this study makes important contributions to urban and demographic literature and highlights several implications for the study of Millennials. First, our study updates prior work by documenting age-specific neighborhood diversity and segregation trends since 2010. Second, studies of increased diversity, on the one hand, and continued resilience in segregation, on the other, have largely focused on older generations or on the population as a whole. Our study is one of the first to examine neighborhood segregation and diversity patterns for young adults across generations. This is an important stage in the life course given that many young adults are old enough to participate directly in the movements impelled by social change, but not old enough to have formed a family and become committed to a career and residence (Rosenfeld, 2006). Finally, our study further builds on the literature highlighting the unique differences of Millennials to prior generations. Understanding the residential patterns of Millennial young adults under the backdrop of broader demographic change has implications for theory and policies, such as those addressing affordable housing, neighborhood change, and urban inequality, more broadly. Our findings show that while Millennials’ residential settings corresponded with prior generations in some ways, it diverged in others, with this divergence indicating a bridge to greater racial diversity and lower segregation. The strength of this bridge, and whether it remains standing at all, will depend on the migration patterns of Millennials as they age, and the neighborhood attainment patterns of future young adult cohorts.

## Figures and Tables

**Fig. A6:**
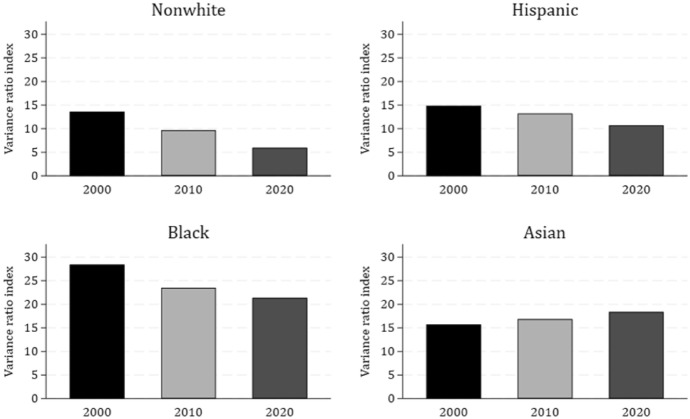
Segregation (variance ratio index) between 25–29-year-old whites and total population (by year). *Note*: Segregation is measured using the variance ratio index (scaled 0–100). White, Black, and Asian are all non-Hispanic. n = 223 MSAs. Young adults during the years represent the GenX (2000), Early Millennial (2010) and Late Millennial (2020) generations

**Fig. A7:**
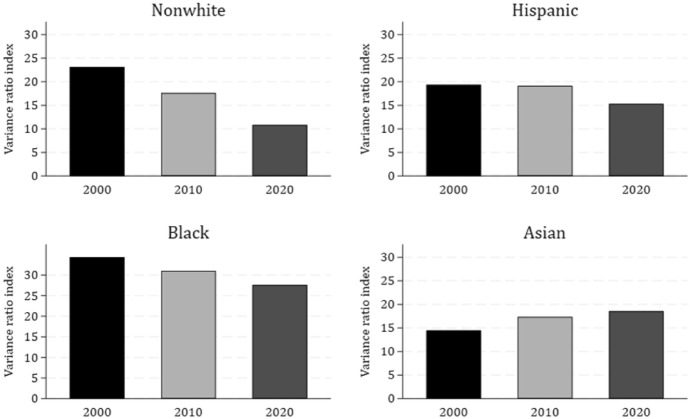
Segregation (variance ratio index) between 35–44-year-old whites and total population (by year). *Note*: Segregation is measured using the variance ratio index (scaled 0–100). White, Black, and Asian are all non-Hispanic. n = 223 MSAs

**Fig. A8:**
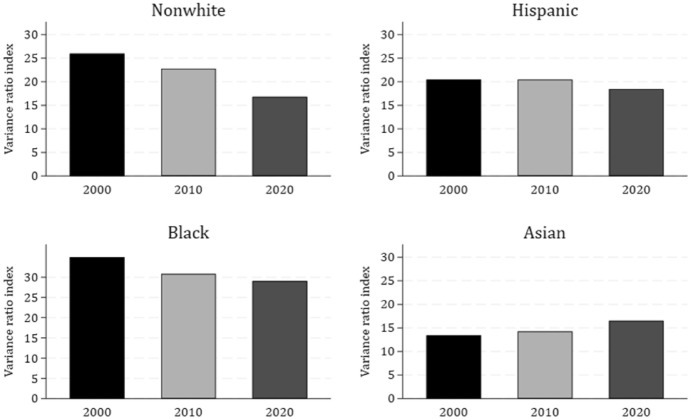
Segregation (variance ratio index) between 45–64-year-old whites and total population (by year). *Note*: Segregation is measured using the variance ratio index (scaled 0–1). White, Black, and Asian are all non-Hispanic. n = 223 MSAs

**Fig. A9:**
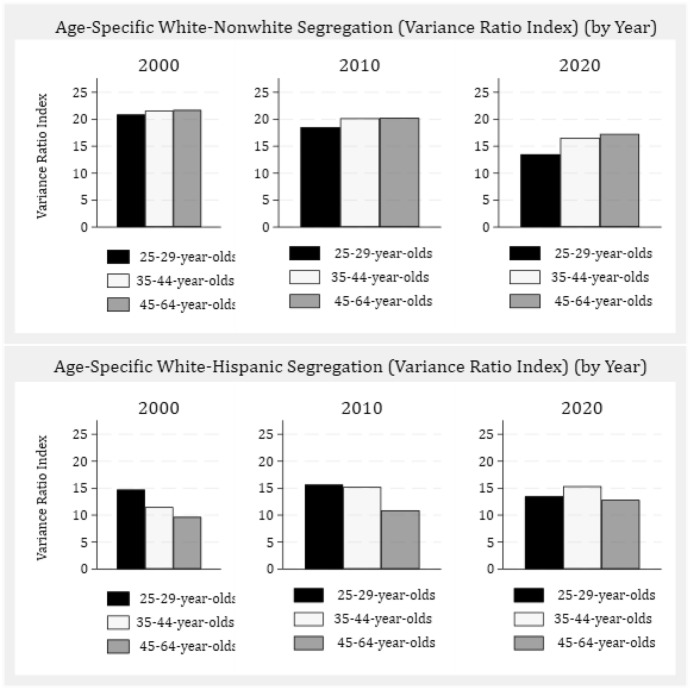
Age-specific white-nonwhite and white-hispanic segregation (variance ratio index), 2000–2020. *Note*: Segregation is measured using the variance ratio index (scaled 0–100). n = 223 MSAs. Young adults during the years represent the GenX (2000), Early Millennial (2010) and Late Millennial (2020) generations

## Appendix

See Tables A1, A2, A3, A4, A5, A6, A7, A8.

**Table A1:** Fixed-effects regression estimates of neighborhood diversity by total age group and year

Variable	35–44		45–64	
	Coef	*p*	Coef	*p*
% 35–44-year-old	− 0.24	0.02		
	(0.11)			
% 45–64-year-old			− 0.63	0.00
			(0.10)	
**Year**				
2000	− 1.01	0.51	6.83	0.00
	(1.53)		(1.36)	
2010	2.75	0.06	5.89	0.00
	(1.46)		(1.64)	
2020	16.29	0.00	24.02	0.00
	(2.00)		(1.92)	
**Year x age group**				
2000	0.69	0.00	0.11	0.02
	(0.10)		(0.05)	
2010	1.00	0.00	0.42	0.00
	(0.10)		(0.07)	
2020	1.11	0.00	0.31	0.00
	(0.13)		(0.08)	
% Nonwhite	20.64	0.00	20.56	0.00
	(2.52)		(2.47)	
Log population	1.35	0.00	1.42	0.00
	(0.24)		(0.25)	
% poor	− 20.08	0.00	− 23.31	0.00
	(3.63)		(3.55)	
% public transit	− 26.05	0.01	− 23.83	0.02
	(9.64)		(9.81)	
% bachelor’s degree	1.69	0.45	− 0.63	0.78
	(2.22)		(2.20)	
% employed in professional	2.37	0.36	9.36	0.00
	(2.61)		(1.98)	
% renter	6.33	0.02	3.36	0.13
	(2.60)		(2.23)	
% residents moved in past 10 yrs	47.58	0.00	44.68	0.00
	(5.14)		(5.23)	
% foreign born	25.91	0.02	26.88	0.02
	(11.27)		(11.31)	
% housing built < 10 yrs	− 20.16	0.00	− 21.13	0.00
	(2.55)		(2.53)	
Central city	3.52	0.00	3.35	0.00
	(0.68)		(0.74)	

MSA clustered standard errors in parentheses. Limited to tracts in metropolitan areas with 1000 racial and ethnic group members in all years (n = 223). Model includes MSA fixed effects and a MSA-specific linear trend

**Table A2:** Fixed-effects regression estimates of neighborhood diversity by White age group and year

Variable	35–44		45–64	
	Coef	*p*	Coef	*p*
% 35–44-year-old	0.05	0.61		
	(0.10)			
% 45–64-year-old			− 0.45	0.00
			(0.06)	
**Year**				
2010	2.57	0.00	0.83	0.19
	(0.65)		(0.64)	
2020	17.85	0.00	17.51	0.00
	(1.49)		(1.35)	
**Year x age group**				
2000	0.48	0.00	0.28	0.00
	(0.06)		(0.02)	
2010	0.79	0.00	0.27	0.00
	(0.08)		(0.03)	
% Nonwhite	25.42	0.00	13.63	0.00
	(2.78)		(3.10)	
Log population	1.66	0.00	1.70	0.00
	(0.27)		(0.30)	
% poor	− 20.66	0.00	− 22.48	0.00
	(3.46)		(3.40)	
% public transit	− 28.49	0.00	− 26.90	0.01
	(9.84)		(9.84)	
% bachelor’s degree	− 2.41	0.27	− 1.74	0.43
	(2.17)		(2.19)	
% employed in professional	11.38	0.00	11.36	0.00
	(2.72)		(2.66)	
% renter	5.29	0.04	3.93	0.10
	(2.51)		(2.41)	
% residents moved in past 10 yrs	49.92	0.00	49.60	0.00
	(5.10)		(5.08)	
% foreign born	27.93	0.01	27.32	0.01
	(11.16)		(11.09)	
% housing built < 10 yrs	− 18.58	0.00	− 19.23	0.00
	(2.63)		(2.68)	
Central city	3.82	0.00	3.77	0.00
	(0.72)		(0.73)	

MSA clustered standard errors in parentheses. Limited to tracts in metropolitan areas with 1000 racial and ethnic group members in all years (n = 223). Model includes MSA fixed effects and a MSA-specific linear trend

**Table A3:** Neighborhood racial segregation between white older age cohorts and total population, 2000–2020

Measure	Year						Δ 2000–2020
	2000		2010		2020		
	Mean	SD	Mean	SD	Mean	SD	
**White 35–44**							
Total Nonwhite	20.91	*9.82*	17.393	*7.92*	12.17	*5.65*	− 8.75
Total Hispanic	17.56	*9.72*	16.996	*8.35*	13.90	*6.90*	− 3.66
Total Black	31.52	*14.52*	27.847	*12.82*	24.50	*11.17*	− 7.01
Total Asian	15.81	*7.40*	16.458	*7.79*	16.61	*7.75*	0.80
**White 45–64**							
Total Nonwhite	22.55	*9.25*	19.689	*7.68*	15.06	*6.08*	− 7.49
Total Hispanic	18.91	*9.69*	18.271	*8.36*	16.01	*7.41*	− 2.90
Total Black	32.70	*13.84*	28.746	*12.40*	26.52	*11.23*	− 6.18
Total Asian	15.96	*7.00*	16.106	*7.39*	17.07	*7.63*	1.11
**Total White (all ages)**							
Total Nonwhite	20.28	*10.57*	18.050	*8.84*	14.41	*7.14*	− 5.87
Total Hispanic	14.74	*9.83*	15.313	*9.10*	13.99	*8.26*	− 0.75
Total Black	27.65	*15.09*	24.621	*13.52*	22.60	*12.08*	− 5.05
Total Asian	10.97	*5.51*	11.553	*5.86*	12.51	*6.35*	1.54

n = 223 MSAs; Segregation is measured by Theil’s Entropy (*H*) (scaled from 0–100); White, Black, and Asian are all non-Hispanic

**Table A4:** Age-specific Neighborhood Racial Segregation, 2000–2020

*Age-specific measure*	Year						Δ 2000–2020
	2000		2010		2020		
	Mean	SD	Mean	SD	Mean	SD	
**35–44 years**							
White-Nonwhite	19.77	*10.41*	17.75	*8.48*	13.92	*6.80*	− 5.85
White-Hispanic	14.03	*9.16*	15.89	*9.01*	14.86	*8.25*	0.84
**45–64 years**							
White-Nonwhite	20.39	*11.16*	18.48	*9.58*	14.78	*7.54*	− 5.62
White-Hispanic	13.07	*9.56*	13.19	*8.90*	13.63	*8.47*	0.56
**65 and up**							
White-Nonwhite	23.46	*13.22*	20.76	*11.28*	15.78	*8.88*	− 7.68
White-Hispanic	13.42	*8.86*	13.13	*9.17*	12.29	*8.32*	− 1.13

n = 223 MSAs; Segregation is measured by Theil’s Entropy (*H*) (scaled from 0–100); White refers to non-Hispanic White

**Table A5:** Mean MSA Racial Segregation (Variance Ratio Index) of Total Population, 2000–2020

*Measure*	Year			Δ 2000–2020
	2000	2010	2020	
White-Nonwhite	22.54	20.70	17.23	− 5.31
White-Hispanic	12.79	14.72	14.41	1.62
White-Black	25.33	22.82	21.02	− 4.31
White-Asian	5.09	6.37	7.85	2.76

n = 223 MSAs; Segregation is measured by the variance ratio index (scaled from 0–100); White, Black, and Asian are all non-Hispanic

**Table A6:** Neighborhood Racial Segregation between White Age Cohorts and Total Population, 2000–2020 (Largest MSAs Only)

Measure	Year						Δ 2000–2020
	2000		2010		2020		
	Mean	SD	Mean	SD	Mean	SD	
**White 25–29**							
Total Nonwhite	0.177	*0.087*	0.140	0.065	0.105	*0.050*	− 0.072
Total Hispanic	0.160	*0.088*	0.148	*0.073*	0.129	*0.065*	− 0.031
Total Black	0.299	*0.139*	0.249	0.117	0.224	*0.105*	− 0.075
Total Asian	0.151	*0.061*	0.155	0.061	0.165	*0.068*	0.015
**White 35–44**							
Total Nonwhite	0.231	*0.099*	0.191	*0.078*	0.134	*0.056*	− 0.097
Total Hispanic	0.196	*0.101*	0.190	*0.085*	0.156	*0.070*	− 0.040
Total Black	0.350	*0.145*	0.310	*0.126*	0.271	*0.111*	− 0.080
Total Asian	0.158	*0.063*	0.166	*0.064*	0.169	*0.068*	0.011
**White 45–64**							
Total Nonwhite	0.245	*0.094*	0.213	*0.077*	0.163	*0.062*	− 0.082
Total Hispanic	0.206	*0.100*	0.200	*0.086*	0.176	*0.077*	− 0.030
Total Black	0.358	*0.138*	0.317	*0.124*	0.291	*0.113*	− 0.068
Total Asian	0.157	*0.060*	0.160	*0.060*	0.170	*0.065*	0.013
**Total White (all ages)**							
Total Nonwhite	0.230	*0.104*	0.204	*0.086*	0.162	*0.070*	− 0.068
Total Hispanic	0.315	*0.150*	0.282	*0.134*	0.256	*0.121*	− 0.059
Total Black	0.168	*0.101*	0.175	*0.091*	0.160	*0.083*	− 0.008
Total Asian	0.115	*0.054*	0.121	*0.055*	0.131	*0.059*	0.017

Sample restricted to most populous MSAs (n = 150); Segregation is measured by Theil's Entropy (H), scaled 0 to 1; White, Black, and Asian are all non-Hispanic. Young adults during the years represent the Baby Boomer (1990), GenX (2000), Early Millennial (2010) and Late Millennial (2020) generations

**Table A7:** Age-specific neighborhood racial segregation, 2000–2020 (largest MSAs only)

Age-specific measure	Year						Δ 2000–2020
	2000		2010		2020		
	Mean	SD	Mean	SD	Mean	SD	
**25–29 years**							
White-Nonwhite	0.210	*0.095*	0.181	*0.080*	0.128	*0.063*	− 0.082
White-Hispanic	0.185	*0.094*	0.179	*0.086*	0.147	*0.079*	− 0.038
**35–44 years**							
White-Nonwhite	0.223	*0.105*	0.198	*0.083*	0.156	*0.066*	− 0.067
White-Hispanic	0.159	*0.095*	0.180	*0.090*	0.169	*0.082*	0.011
**45–64 years**							
White-Nonwhite	0.231	*0.112*	0.208	*0.096*	0.166	*0.076*	− 0.065
White-Hispanic	0.148	*0.100*	0.149	*0.092*	0.155	*0.087*	0.007
**65 and up**							
White-Nonwhite	0.267	*0.131*	0.235	*0.111*	0.180	*0.088*	− 0.087
White-Hispanic	0.148	*0.091*	0.146	*0.094*	0.139	*0.085*	− 0.009

Sample restricted to most populous MSAs (n = 150); Segregation is measured by Theil's Entropy (H), scaled 0 to 1; White refers non-Hispanic White. Young adults during the years represent the Baby Boomer (1990), GenX (2000), Early Millennial (2010) and Late Millennial (2020) generations

**Table A8:** Neighborhood racial segregation between white age cohorts and total population in high and low proportion Asian areas, 2000–2020

Measure	Year						Δ 2000–2020
	2000		2010		2020		
	Mean	SD	Mean	SD	Mean	SD	
**High Asian**							
*White 25–29*							
Total nonwhite	0.122	*0.080*	0.109	0.066	0.088	*0.053*	− 0.035
Total Asian	0.149	*0.064*	0.149	0.076	0.154	*0.084*	0.004
*White 35–44*							
Total nonwhite	0.164	*0.096*	0.143	*0.081*	0.106	*0.059*	− 0.058
Total Asian	0.163	*0.071*	0.165	*0.081*	0.163	*0.088*	0.000
*White 45–64*							
Total nonwhite	0.186	*0.092*	0.173	*0.083*	0.136	*0.067*	− 0.050
Total Asian	0.169	*0.071*	0.174	*0.080*	0.177	*0.086*	0.008
*Total White (all ages)*							
Total nonwhite	0.174	*0.102*	0.167	*0.091*	0.140	*0.077*	− 0.034
Total Asian	0.130	*0.067*	0.139	*0.075*	0.147	*0.081*	0.017
**Low Asian**							
*White 25–29*							
Total nonwhite	0.151	*0.082*	0.123	*0.063*	0.083	*0.044*	− 0.068
Total Asian	0.176	*0.053*	0.149	*0.040*	0.153	*0.044*	− 0.022
*White 35–44*							
Total Nonwhite	0.183	*0.073*	0.170	*0.067*	0.105	*0.044*	− 0.078
Total Asian	0.155	*0.028*	0.158	*0.039*	0.141	*0.035*	− 0.014
*White 45–64*							
Total nonwhite	0.176	*0.062*	0.167	*0.051*	0.120	*0.040*	− 0.056
Total Asian	0.146	*0.018*	0.138	*0.026*	0.135	*0.032*	− 0.011
*Total White (all ages)*							
Total nonwhite	0.161	*0.077*	0.153	*0.062*	0.112	*0.052*	− 0.049
Total Asian	0.114	*0.022*	0.108	*0.025*	0.103	*0.026*	− 0.011

Segregation is measured by Theil's Entropy (H), scaled 0 to 1; *High Asian* (n = 49) includes all MSAs located in the six states with the largest proportion of Asian residents in 2010 (Hawaii, California, New York, New Jersey, Nevada, and Washington). *Low Asian* (n = 5) includes all MSAs located in the five states with the lowest proportion of Asian residents in 2010 (Wyoming, Montana, West Virginia, Maine, and Mississippi). Young adults during the years represent the Baby Boomer (1990), GenX (2000), Early Millennial (2010) and Late Millennial (2020) generations

See Figs. A1, A2, A3, A4, A5, A6, A7, A8, and A9.

## Footnotes

1 The original decennial census release in 2000 and 2010 did not report race/ethnicity-by-age data for the entire population (e.g., non-Hispanic Black; non-Hispanic Asian; etc.), nor did the preliminary release of the 2020 decennial census. However, IPUMS-NHGIS, in collaboration with census administrators, introduced and released an experimental data product (called “Differentially Private 2010 Data” or “Privacy-Protected 2010 Census Demonstration Data”) in 2023 that provided additional counts for the remaining race-by-Hispanic groups made possible by disclosure procedures that involved strategically injecting noise into all subgroup population counts—what census documentation called “differential privacy” disclosure frameworks. The publicly available privacy-protected summary files produced via this demonstration product draw on underlying, untabulated microdata files (also privacy protected) for the entire nation. This privacy protection procedure in 2020 used a different disclosure avoidance system (DAS) compared to the initial procedures employed in 2010 and 2000 both in how noise is injected into the data, as well as in the minimum threshold applied to each racial/ethnic group’s tract population for disclosure eligibility (i.e., in 2020 there were no minimum population thresholds, but age-race counts were not disclosed for a substantial number of tracts in 2000 and 2010 due to low subgroup populations). To limit statistical bias, we restrict our analysis to the counts for Non-Hispanic White and Hispanic groups, which were included in the initial decennial census release.

2 Disaggregated age by race by ethnicity counts are only available for a subset of the population in 1990, namely, the under-18 Hispanic, non-Hispanic White and non-Hispanic nonwhite population. Race-by-age counts *are* available in 1990 for other groups, but they did not disaggregate by Hispanicity (e.g., age-disaggregated counts for Black residents include those that are non-Hispanic and Hispanic, etc.).

3 For our decomposition, we aggregate population counts for only these age categories (i.e., we exclude the 18–24 and 30–34 populations). We chose these categories to ensure that we measured a distinct category of young adults that mapped onto our generations while minimizing overlap between these cohorts in any year. Sensitivity analyses including population counts for all age categories generate the same substantive patterns.

4 We do experience some generational overlap, albeit minimal, for the 45- to 64-year-old age group in 2020. The earliest Gen Xers and late Baby Boomers fall within this age range in 2020, but the proportion of Boomers comprising this age group is much higher.

5 The variance ratio index, also known as the separation index, the squared coefficient of variation, and eta squared (η2) (Fossett, 2017; Rich et al., 2021), is another decomposable and structural measure of segregation that captures how evenly racial/ethnic groups are distributed among neighborhoods within MSAs. When measured on a scale from 0 to 1, a value of 0 would connote a completely even distribution across the MSA, such that neither focal group overconcentrates in any subset of neighborhoods (given the racial composition of the MSA). On the other hand, higher values would indicate increasing racial polarization, such that the focal groups of interest populate into entirely separately neighborhoods. In the appendix, we present supplementary results from parallel models (Appendix Figures A6–A9).

6 We note that segregation estimates involving the Asian population may be more unstable compared to other groups due to their uneven, and sometimes much smaller representation within and across MSAs. As a robustness check, we performed a sub-analysis on MSAs located in states with the highest and lowest proportion of Asian residents (see Appendix Table A8) and our overall results held. Nonetheless, as a conservative view, we present our findings for White-Asian segregation as illustrating general trends rather than precise estimates.
